# Targeting PFKFB3 to restore glucose metabolism in acute pancreatitis via nanovesicle delivery

**DOI:** 10.1186/s10020-025-01261-y

**Published:** 2025-07-05

**Authors:** Hai Jiang, Zhipeng Xu, Qi Song, Junjie Tao, Jia Liu, Qiang Wang, Huaisheng Zhang, Heng Zhu, Qiliang Li, Lei Li

**Affiliations:** Department of Emergency Surgery, the First Affiliated Hospital of Bengbu Medical University, No. 287, Changhuai Road, Bengbu, 233000 Anhui Province China

**Keywords:** Acute pancreatitis, Glucose metabolism disorder, PFKFB3 inhibitor, Nanovesicles, Single-cell RNA sequencing, Machine learning

## Abstract

**Background:**

Acute pancreatitis (AP) is a severe inflammatory disease frequently accompanied by disturbances in glucose metabolism, which further complicate the disease prognosis. This study aims to explore the role of PFKFB3, a key glycolytic enzyme, in regulating glucose metabolism in AP and assess the potential of PFKFB3 inhibition via nanovesicle delivery to mitigate metabolic dysfunction.

**Methods:**

Transcriptomic data from Gene Expression Omnibus (GEO), including single-cell RNA sequencing (scRNA-seq) and bulk RNA sequencing, were analyzed to investigate the molecular mechanisms involved in glucose metabolism dysregulation in AP. The therapeutic effects of PFKFB3 inhibition via nanovesicle-based delivery were evaluated using both in vivo and in vitro AP models.

**Results:**

PFKFB3 inhibition significantly restored normal glycolytic function and improved glucose metabolism in AP models. Moreover, nanovesicle-mediated delivery also alleviated both inflammation and metabolic disturbances, highlighting its promise as a therapeutic strategy for managing glucose dysfunction in AP.

**Conclusion:**

Our findings identify PFKFB3 as a critical therapeutic target for treating glucose metabolism disorders in acute pancreatitis. Nanovesicle-based PFKFB3 inhibition may serve as an innovative approach to address metabolic complications associated with AP, offering a new direction for therapeutic interventions in inflammatory diseases.

**Graphical Abstract:**

Molecular Mechanism of EVs-mediated Delivery of PFKFB3 Inhibitor in Ameliorating Glucose Metabolism Disorder Post-AP.
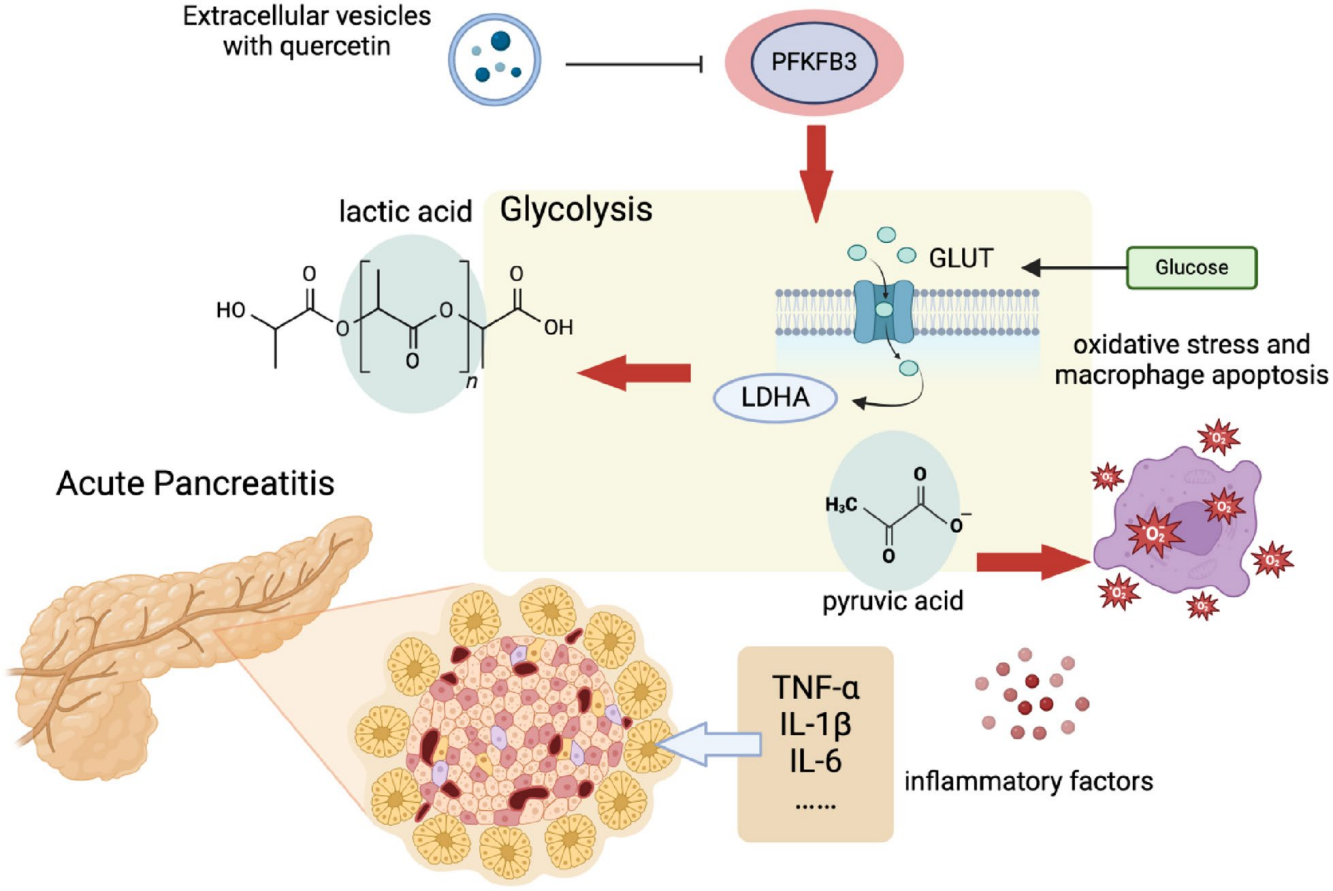

**Supplementary Information:**

The online version contains supplementary material available at 10.1186/s10020-025-01261-y.

## Introduction

Acute pancreatitis (AP) is a common acute inflammatory disease of the digestive system with a complex and diverse pathogenesis (Lin and Huang 2024; Zerem et al. 2023; Qi-Xiang et al. 2022; Del Poggetto et al. 2021). Although most patients with AP recover with appropriate medical intervention, severe cases can lead to serious complications or even death (Zhang and Cui 2024; Liberis et al. 2021; Boxhoorn et al. 2021; Mądro 2022). Notably, the primary causes of AP include gallstones, excessive alcohol consumption, adverse drug reactions, and metabolic disorders (Del Gaudio et al. 2023; Thakur et al. 2023). In addition to acute inflammatory responses, glucose metabolism disorders are a significant feature of AP. These metabolic disturbances not only exacerbate the disease course but also significantly affect the clinical prognosis of patients. Therefore, investigating the relationship between AP and glucose metabolism disorders and exploring effective interventions are crucial for improving patient outcomes (Jiang et al. 2023a, b).

During the exploration of the link between AP and metabolic dysfunction, glycolysis, an essential pathway of intracellular glucose metabolism, plays a pivotal role in maintaining energy supply and metabolic homeostasis (Yang et al. 2024; Fuller and Kim 2021; Padilla and Lee, 2021; Qiu et al. 2021). In various inflammatory diseases, the abnormal activation of the glycolysis pathway is considered a major driver of disease progression (Saadh et al. 2023; Wei et al. 2023; Mastrocola et al. 2022). PFKFB3 (6-phosphofructo-2-kinase/fructose-2,6-bisphosphatase 3), a key regulatory enzyme in the glycolysis pathway, has garnered attention for its role in AP (Jiang et al. 2023a, b; Sun et al. 2023; Yang et al. 2023). Studies have shown that PFKFB3 activity is closely related to the progression of various metabolic and inflammatory diseases, but its specific mechanism in AP remains unclear. For instance, PFKFB3 may modulate AP severity by controlling acinar cell-mediated Ca²⁺ overload (Zhang et al. 2024a, b), and may prevent the progression of severe AP via the Nrf2/HO-1 signaling pathway in macrophages (Ergashev et al. 2024). However, the precise mechanisms by which PFKFB3 regulates AP remain unclear (Xiao et al. 2023; Almeida et al. 2023; Jones et al. 2022). Accordingly, regulating PFKFB3, particularly through small molecule inhibitors, offers a promising strategy for improving glucose metabolism disorders in AP (Lu et al. 2021; Selinger et al. 2022).

With the development of bioinformatics and high-throughput sequencing technologies, researchers can access large amounts of transcriptome data from public databases and systematically analyze them using bioinformatics tools to reveal changes in disease-related gene expression and signaling pathways (Micheel et al. 2021; Erkes et al. 2023). In this study, we utilized AP-related transcriptome datasets from the Gene Expression Omnibus (GEO) database, including bulk RNA sequencing (bulk RNA-seq) and scRNA-seq data. We applied methods such as Gene Set Enrichment Analysis (GSEA) and Weighted Gene Co-expression Network Analysis (WGCNA) for data integration and analysis to identify significantly altered metabolic and immune pathways in AP. These analytical methods allowed us to screen for key genes closely associated with the pathological processes of AP and further explore their roles in disease progression (Yuan et al. 2025; Trubetskoy et al. 2022; Guo et al. 2021a, b, c; Moran et al. 2023).

Meanwhile, in recent years, cell membrane nanovesicles have gained significant attention as drug delivery systems (Guo et al. 2024; Liang et al. 2021; Ou et al. 2023; Feng et al. 2022). Due to their unique biocompatibility and targeting capabilities, cell membrane nanovesicles are considered ideal carriers for drug delivery (Lopes et al. 2022; Ou et al. 2023; Prasad et al. 2023). In our study, extracellular vesicles (EVs) were isolated from AR42 J pancreatic acinar cells, a rat-derived exocrine pancreatic cell line with typical acinar cell characteristics. These cells are widely used to model AP-related pathological changes, including suppressed proliferation and enhanced autophagy (Guo et al. 2021a, b, c; Zhao et al. 2021). Furthermore, AR42 J cells exhibit strong secretory activity and produce abundant EVs containing biologically active substances relevant to pancreatic function and AP progression (Wen et al. 2021; Guo et al. 2021a, b, c). Thus, we selected AR42 J-derived EVs for downstream experiments. Loading the PFKFB3 small molecule inhibitor quercetin into cell membrane nanovesicles using electroporation can significantly enhance the drug’s stability and targeting in vivo (Nain et al. 2021; Tumova et al. 2019). Quercetin was chosen due to its known antioxidant, anti-inflammatory, and immunomodulatory properties (Sun et al. 2017). Previous studies have demonstrated its ability to attenuate inflammatory responses in AP (Zhan et al. 2021). However, quercetin’s low solubility limits its efficacy when administered orally (Zarenezhad et al. 2023). Encapsulating quercetin in nanovesicles can enhance its solubility and bioavailability, improve drug stability and sustained release, and facilitate targeted delivery to the site of inflammation, thereby improving therapeutic efficacy. In this study, we successfully loaded quercetin into the EVs derived from AR42 J pancreatic acinar cells and validated its effects on improving glucose metabolism disorders following AP through a series of in vitro and in vivo experiments. The in vitro LPS-induced inflammation model in RAW264.7 cells demonstrated that quercetin-loaded extracellular vesicles (Q-Ex) significantly reduced PFKFB3 expression and the release of pro-inflammatory cytokines, inhibited the glycolysis process, thereby enhancing macrophage viability and inhibiting apoptosis.

In summary, the aim of this study was to investigate the role and potential mechanisms of a PFKFB3 small molecule inhibitor loaded in cell membrane nanovesicles in improving glucose metabolism disorders following AP by integrating systematic bioinformatics analysis and nanovesicle drug delivery technology. By analyzing transcriptome data from the GEO database, we identified significantly altered metabolic and immune pathways in AP and screened for the key gene PFKFB3. Subsequently, using nanovesicle technology, we successfully loaded the PFKFB3 small molecule inhibitor quercetin into cell membrane nanovesicles and validated its efficacy in both in vitro and in vivo experiments. This innovative therapeutic strategy holds promise for providing more effective treatment options for patients with AP, improving clinical outcomes, and enhancing quality of life.

## Materials and methods

### Data acquisition and preprocessing

To investigate the transcriptome of AP, relevant datasets were downloaded from the GEO database (https://www.ncbi.nlm.nih.gov/geo/), including peripheral blood samples from 87 AP patients and 32 healthy controls.

The single-cell RNA sequencing (scRNA-seq) dataset GSE250486 was obtained, which included samples from two normal mouse pancreatic tissues (GSM7979747 and GSM7979748) and two AP mouse pancreatic tissues (GSM7979746 and GSM7979749).

As these data were sourced from public databases, ethical approval or informed consent was not required (Barrett et al. 2013).

### GSEA

Version 3.0 of the GSEA software was downloaded from the official GSEA website. The c7.immunesigdb.v7.4.symbols.gmt subset was obtained from the Molecular Signatures Database to evaluate relevant pathways and molecular mechanisms. Based on gene expression profiles and phenotype grouping, the minimum and maximum gene set sizes were set to 5 and 5000, respectively, and 1000 permutations were performed. A *p*-value of < 0.05 and a false discovery rate (FDR) of < 0.25 were considered statistically significant.

Additionally, the c2.cp.kegg.v7.4.symbols.gmt subset was also downloaded from the Molecular Signatures Database (http://www.gsea-msigdb.org/gsea/downloads.jsp) for further pathway evaluation under the same settings (Subramanian et al. 2005).

### xCell immune infiltration analysis

The xCell algorithm was applied to assess the infiltration levels of 64 types of immune and stromal cells, including extracellular matrix cells, epithelial cells, hematopoietic progenitor cells, innate immune cells, and adaptive immune cells.

For immune cell enrichment analysis, the ssGSEA method from the R package GSVA was used. Prior to the analysis, the raw gene expression data from the aforementioned microarray datasets were normalized.

Differences in enrichment scores between the two groups were statistically analyzed, and violin plots were generated using the ggplot2 package in R. A *p*-value of < 0.05 was considered statistically significant. All analyses and visualizations were performed using R version 4.2.1 (Zhong et al. 2024).

### WGCNA

The Median Absolute Deviation (MAD) for each gene was first calculated based on the gene expression profiles, and the top 50% of genes with the lowest MAD values were removed. The goodSamplesGenes function from the R package WGCNA was then used to eliminate outlier genes and samples. A scale-free co-expression network was constructed, with the minimum module size set to 30 and the sensitivity parameter set to 3. Modules with a dissimilarity threshold below 0.25 were merged, resulting in 15 co-expression modules. Genes that could not be assigned to any module were grouped into a grey module.

Pearson correlation tests (*p* < 0.05) were used to evaluate the relationship between module eigengenes and sample traits. The module most significantly associated with AP was selected, and its genes were retained for further analysis (Wang et al. 2023a, b).

### Differential gene expression and combined gene ontology (GO)/Kyoto encyclopedia of genes and genomes (KEGG) enrichment analysis

Based on the GEO dataset, differentially expressed genes (DEGs) were identified using the R package Limma (linear models for microarray data), with selection criteria set at |log₂FoldChange| > 1 and *p*-value < 0.05. The DEGs were subsequently visualized by generating volcano plots and heatmaps using the ggplot2 and pheatmap packages, respectively. All analyses were conducted using R version 4.3.1 (R Foundation for Statistical Computing).

For gene set functional enrichment analysis, GO annotations were obtained from the org.Hs.eg.db package (version 3.1.0) and used as the background reference. The enrichment analysis of gene sets was performed using the clusterProfiler package (version 3.14.3). For KEGG pathway analysis, the most recent gene annotations were retrieved via the KEGG REST API, and genes were mapped accordingly. Enrichment analysis was also conducted using clusterProfiler under the same settings. In both analyses, the minimum and maximum gene set sizes were set to 5 and 5000, respectively, and results with *p*-values < 0.05 and false discovery rate (FDR) < 0.25 were considered statistically significant. Final data visualizations were carried out using ggplot2 (Wang et al. 2017).

### Key factor identification

To identify key factors closely associated with AP, an intersection analysis was performed between the DEGs and the key module genes derived from WGCNA. Venn diagrams were generated using the VennDiagram package (version 1.6.20) in the R environment to visually present the overlap between the two gene sets (Ye and Kong 2025).

### Correlation pie chart

The correlations among variables were evaluated using pairwise Spearman correlation coefficients. The correlation strengths were interpreted as follows: |*r*| > 0.95 indicated a significant correlation; |*r*| ≥ 0.8 indicated a high correlation; 0.5 ≤ |*r*| < 0.8 indicated a moderate correlation; 0.3 ≤ |*r*| < 0.5 indicated a low correlation; and |*r*| < 0.3 indicated a weak correlation. The results of the correlation analysis were visualized as pie charts using the ggplot2 package (Dessì et al. 2022).

### Receiver operating characteristic (ROC) curve analysis

ROC curve analysis was performed using the pROC package in R, based on the expression values of candidate genes, to evaluate their diagnostic performance in distinguishing disease status. The pROC package was employed to ensure convex ROC curves by automatically correcting the order of the data.

The ROC curves illustrated the relationship between sensitivity and specificity. The x-axis represented 1 minus specificity (i.e., the false positive rate), where values closer to zero indicated higher specificity. The y-axis represented sensitivity (true positive rate), with higher values indicating better sensitivity.

The area under the ROC curve (AUC) was used to assess diagnostic accuracy. AUC values ranged from 0.5 to 1, with values closer to 1 indicating stronger diagnostic performance (Efgan et al. 2024).

### ScRNA-seq data analysis and cell clustering

Data quality control was performed based on the following criteria: 200 < nFeature_RNA < 5000 and percent.mt < 20. We selected the top 2000 highly variable genes based on variance for further analysis.

To reduce the dimensionality of the scRNA-seq dataset, principal component analysis (PCA) was performed on the expression of the top 2000 highly variable genes. Using the ElbowPlot function in the Seurat package, the top 20 principal components (PCs) were selected for downstream analysis. Major cell subpopulations were identified using the FindClusters function in Seurat with the resolution set to the default value (res = 1). The UMAP algorithm was then applied to reduce the nonlinear dimensionality of the scRNA-seq data. Marker genes for each cell subpopulation were identified using the Seurat package and annotated using known lineage-specific marker genes with the assistance of the online tool CellMarker. Finally, cell-to-cell communication analysis was conducted using the CellChat package in R (Zhang et al. 2024a, b).

DEGs in the scRNA-seq dataset were identified using the Limma package in R, with a *p*-value < 0.05 set as the threshold for significant differences between samples.

### Cell culture

AR42 J (CRL-1492, ATCC, USA) and RAW264.7 (SC-6005, ATCC, USA) cell lines were cultured in RPIM-1640 (11875093, Thermo Fisher) and high-glucose DMEM (11965092, Thermo Fisher) media, respectively. The media were supplemented with 10% fetal bovine serum (A5669701, Thermo Fisher) and 1% penicillin/streptomycin (15070063, Thermo Fisher). Cells were maintained in a 37 °C incubator with 5% CO_2_ to promote stable growth (Zhou et al. 2025).

### EVs isolation and purification

When AR42 J cells reached 70–80% confluency in culture dishes, the culture medium was collected. The collected medium was centrifuged at 2000×g for 30 min with a centrifuge radius of 11 cm to remove cell debris and apoptotic bodies. The supernatant was retained, and an EVs reagent (4478359, Invitrogen, CA) equal to 0.5 volume of the supernatant was added. The mixture was incubated overnight at 4 °C. Subsequently, the samples were centrifuged at 10,000×g for 60 min at 4 °C with a centrifuge radius of 11 cm, and the supernatant was discarded. Finally, the samples were resuspended in PBS, aliquoted, and stored at − 80 °C (Li et al. 2022).

### Fluorescent labeling and loading of PFKFB3 small molecule inhibitor

A total of 150 µL of EVs solution (1 mg/mL) was incubated with 50 µL of the PFKFB3 small molecule inhibitor quercetin (Quercetin, SM2147, Beyotime Biotechnology; 1 mg/mL) in an electroporation cuvette. The mixture was subjected to electroporation at 350 V for 150 milliseconds using a Bio-Rad electroporator (USA).

After electroporation, the mixture was incubated at 37 °C for 30 min to allow complete closure of the membrane pores. The resulting solution was then transferred into a 100 kDa ultrafiltration tube and centrifuged at 4000×g for 30 min to remove any unloaded quercetin. The Q-Ex were collected for further use (Pomatto et al. 2019).

### Transmission electron microscopy (TEM)

The morphology of the EVs was evaluated using a TEM (JEM-2100, JEOL, Japan). Freshly prepared EVs were stained with 3% phosphotungstic acid (P4006, Sigma-Aldrich) for 5 min and then placed on a copper grid to dry at 65 °C. Finally, the EVs were observed under the TEM at a magnification of 70,000x (Javadi et al. 2024).

### Dynamic light scattering (DLS) analysis

The size distribution of Q-Ex re-dispersed in distilled water was assessed using a Zetasizer Nano ZS (Malvern Instruments, UK). The nanoparticle samples were diluted to an appropriate concentration at 25 °C, and measurements were repeated three times to ensure accuracy (Lyu et al. 2021).

### HPLC analysis of quercetin solubility

The solubility of quercetin in EVs was determined using high-performance liquid chromatography (HPLC). First, EVs were lysed using a lysis buffer containing Triton X-100 (P0013 J, Beyotime Biotechnology). Following the methods outlined in previous research, the concentration of quercetin in the solution was detected using a Waters Alliance 2695–2487 HPLC system equipped with a Waters Symmetry C18 column. The detection wavelength was set to 280 nm. During chromatographic separation, gradient elution was performed with mobile phase A (H_2_O) and mobile phase B (acetonitrile containing 0.1% trifluoroacetic acid) at a flow rate of 1 mL/min. The UV detector was set to a wavelength of 280 nm, and data were analyzed using Empower software (Zhuang et al. 2023).

### Cellular uptake of Q-Ex

To track the uptake of Q-Ex within cells, quercetin was labeled with Cy5.5 (ab6955, Abcam). The procedure was as follows: Cy5.5 was dissolved in DMSO to prepare a 10 mM stock solution. It was then reacted with quercetin in an appropriate buffer at pH 7.0–8.5. After completion of the reaction, unreacted Cy5.5 was removed by dialysis or gel filtration to obtain Q-Ex with a final density of 1 g/mL. RAW264.7 cells were cultured in 6-well plates (4 × 10^5^ cells per well) for 24 h until the cells reached 70% confluency. Q-Ex (density: 1 g/mL) was added to the cell culture medium and incubated for 1 h. The cells were then washed with PBS to remove any free quercetin. Finally, fluorescence intensity was monitored using a fluorescence microscope (Carl Zeiss Meditec AG, Jena, Germany) at 680/710 nm (Zhang et al. 2019).

### Cell grouping

RAW264.7 cells were used to simulate an inflammatory environment in vitro with the following groups: DMSO + PBS group: Control group treated with 45 µL DMSO solution; Q-Ex + PBS group: Drug control group treated with 45 µL Q-Ex for 24 h; DMSO + LPS group: Inflammation model group treated with 45 µL DMSO solution and 500 ng/mL lipopolysaccharide (LPS, SMB00704, Sigma-Aldrich) for 24 h; Q-Ex + LPS group: Treatment group simultaneously treated with 500 ng/mL LPS and 45 µL Q-Ex for 24 h.

To minimize cytotoxicity, the final concentration of DMSO used was less than 0.1%. The concentration of Q-Ex was 1 g/mL, and the final concentration of quercetin was 50 µM with an exposure time of 24 h (Xu et al. 2017).

### CCK-8 assay

Cells were co-cultured in 96-well plates at a density of 4 × 10^3^ cells per well for 0, 12, 24, 36, and 48 h. Before measurement, cells from each group were washed with PBS and then incubated with 100 µL of CCK-8 solution (RM02823, ABclonal, China) at 37 °C for 1 h. Absorbance was measured at 450 nm using a microplate reader (Thermo Fisher Scientific, Inc.). Each independent experiment was repeated three times. Data are presented as the mean ± standard deviation (Guo et al. 2022).

### Flow cytometry analysis of apoptosis

Apoptosis was analyzed using the Annexin V-FITC/PI Apoptosis Detection Kit (CA1020, Solarbio) according to the manufacturer’s instructions. Treated cells were collected and resuspended in a binding buffer, then incubated with Annexin V-FITC and PI dyes for 15 min in the dark at room temperature. The cells were then analyzed using a flow cytometer (BD Biosciences, USA), and the percentage of apoptotic cells was calculated using analysis software (Zhang et al. 2022).

### TUNEL assay

The TUNEL assay was performed using the TUNEL Apoptosis Detection Kit (C1091, Beyotime) according to the manufacturer’s instructions. First, the samples were incubated at room temperature for 5 min with immunostaining permeabilization solution, then washed with PBS. Next, they were incubated at room temperature for 20 min with an endogenous peroxidase-blocking solution and washed again with PBS. The samples were then incubated at 37 °C in the dark for 60 min with an appropriate amount of biotin-labeled solution, followed by a PBS wash. Afterward, the labeling reaction stop solution was added, and the samples were incubated at room temperature for 10 min and washed with PBS. Subsequently, streptavidin-HRP working solution and DAB staining solution were added. Finally, the samples were mounted with an anti-fade mounting medium containing DAPI and observed and imaged using a fluorescence microscope (IX73, Olympus) (Chen et al. 2024).

### Cell metabolism testing

Cell metabolism was assessed using an Agilent Seahorse XF96 Analyzer (103179-100, Agilent Technologies) according to the manufacturer’s protocol to determine and analyze the oxygen consumption rate (OCR) and extracellular acidification rate (ECAR). Cells from each group were seeded into the wells of a cell culture microplate.

For OCR measurement, cells were sequentially treated with oligomycin (10^−3^ mM), trifluoromethoxy carbonyl cyanide phenylhydrazone (FCCP) (10^−3^ mM), and rotenone/antimycin A (5 × 10^−4^ mM) from the Seahorse XF Cell Mito Stress Test Kit (103015-100, Agilent). The OCR was recorded using the XF96 Analyzer.

For ECAR measurement, cells were sequentially exposed to glucose (10 mM), oligomycin (10^−3^ mM), and 2-deoxy-D-glucose (2-DG; 50 mM) from the Seahorse XF Glycolysis Stress Test Kit (103020-100, Agilent). The ECAR was recorded using the XF96 Analyzer (Cai et al. 2021).

### Reactive oxygen species (ROS) detection

We measured ROS levels in RAW264.7 cells using the ROS Assay Kit (S0033S, Beyotime, Shanghai) following the manufacturer’s instructions. Briefly, cells were seeded in 12-well plates and incubated with a mixture containing 10 µM DCFH-DA for 30 min. After two PBS washes, the cell suspension was filtered through a cell strainer, and the resulting samples were analyzed by flow cytometry (Ma et al. 2018).

### RT-qPCR

Total RNA was extracted from cells using the Trizol reagent kit. cDNA was synthesized from the extracted RNA using a reverse transcription kit (RR047 A, Takara, Japan). The reaction system for RT-qPCR was prepared using the SYBR^®^ Premix Ex TaqTM II kit (DRR081, Takara, Japan), and the samples were subjected to RT-qPCR on a real-time fluorescence quantitative PCR instrument (ABI7500, Thermo Fisher, USA). The PCR program was designed as follows: pre-denaturation at 95 °C for 30 s, followed by 40 cycles of 95 °C for 5 s and 60 °C for 30 s, then an extension at 95 °C for 15 s, 60 °C for 60 s, and another extension at 90 °C for 15 s, followed by the generation of the amplification curve. GAPDH was used as an internal control. All RT-qPCR assays were performed in triplicate wells, and each experiment was repeated three times. The 2^−ΔΔCt^ method was used to represent the fold change in target gene expression between the experimental and control groups. The formula is ΔΔCt = ΔCt _(experimental group)_ - ΔCt _(control group)_, where ΔCt = Ct _(target gene)_ - Ct _(internal control gene)_. Ct is the number of amplification cycles required for the fluorescence intensity to reach the threshold, indicating exponential growth of the amplification. Primer sequences are listed in Table S1 (Zhang et al. 2023).

### Establishment and treatment of the AP rat model

Twenty-four healthy male Sprague-Dawley (SD) rats, aged 6–8 weeks, were purchased from Shanghai Slack Laboratory Animal Co., Ltd. The rats were housed in an SPF-grade animal laboratory with separate cages, maintaining a humidity of 60–65% and a temperature of 22 to 25 °C. They had free access to food and water under a 12-hour light/dark cycle. After a one-week acclimatization period, the health status of the rats was observed before starting the experiments. The experimental procedures and animal use protocol were approved by the Animal Ethics Committee.

AP was induced in rats by intraperitoneal injections of cerulein (SY0466, Beijing Baiao Leibo Technology Co., Ltd., Beijing, China) at a dose of 50 µg/kg. The injections were administered every hour for a total of seven injections. The control group received an equal volume of normal saline via intraperitoneal injection. Prior to injection, the rats were fixed in a supine position, and the injection site was to the right of the midline of the lower abdomen. The needle was inserted at approximately a 45° angle to the rat, and care was taken to avoid injecting into blood vessels, the bladder, or intestines by checking for the absence of blood or intestinal fluid upon aspiration. The rats were divided into four groups: DMSO + NS (normal saline) group, Q-Ex + NS group, DMSO + AP group, and Q-Ex + AP group. The Q-Ex treatment was administered via intraperitoneal injection three times: 12 h and 6 h before AP induction, and 6 h after AP induction, at a dose of 50 mg/kg body weight each time (Junyuan et al. 2018; Miao et al. 2019). After the treatments, the rats were euthanized by cervical dislocation, and blood samples were collected from the heart to measure serum cytokine levels. Pancreatic tissues were also collected for subsequent histological examination (Zhao et al. 2019).

### Hematoxylin and Eosin (H&E) staining and scoring

Tissues were paraffin-embedded, deparaffinized, and rehydrated through a gradient series of alcohol. Sections were stained with hematoxylin (#AR-0711, Dingguo Prosperous Biotechnology Co., Ltd.) for 1–2 min, followed by eosin staining (#AR-0731, Dingguo Prosperous Biotechnology Co., Ltd.). After staining, sections were dehydrated through a graded series of alcohol and then mounted. The pancreas was divided into the duodenum (head) and spleen (tail) regions. Each pancreas was examined using a single paraffin-embedded section stained with H&E. A pathologist, blinded to the pancreatitis induction method, scored the tissue for hemorrhage, inflammation, and necrosis across 20 fields of view. The scoring reflected the extent of acinar necrosis, inflammation, and hemorrhage, ranging from 0 (no findings) to 24 (most severe findings) (Zhang et al. 2018).

### Immunohistochemistry staining

Tissue sections were prepared and deparaffinized to water, as described above. The sections were then incubated in a blocking solution (0.1 M PBS containing 0.3% Triton-X and 2% normal serum) for 1 h. Subsequently, primary antibody amylase (ab231119, 1:200, Abcam, USA) was added and incubated overnight at 4 °C. The sections were then washed three times with PBS and incubated with 100 µL of enzyme-labeled goat anti-mouse/rabbit IgG polymer (PV6000, Zhongshan Golden Bridge, China) for 30 min. Following this, the sections were developed using the DAB staining kit (ZLI-9018, Zhongshan Golden Bridge, China). The staining was observed under a microscope, and images were captured. Each group included 6 animals, and 3 sections from each animal were stained and analyzed. For each section, 3 fields of view were selected for imaging. The images were analyzed using ImageJ 1.48u software (version 1.48, National Institutes of Health, USA) to calculate the percentage of the area with positive staining (Zhu et al. 2020).

### Immunofluorescence staining

Tissue sections were prepared and deparaffinized as described above, then incubated in a blocking solution (0.1 M PBS containing 0.3% Triton-X and 2% normal serum) for 1 h. Next, mouse monoclonal antibody against 8-Hydroxy-2’-deoxyguanosine (8-OHdG) (ab48508, 1:250, Abcam, USA) was added, and the sections were incubated overnight at 4 °C. The sections were then washed three times with PBS and incubated with goat anti-mouse IgG (H + L) fluorescent secondary antibody (A0473, Beyotime, China) for 3 h, followed by another three washes with PBS. Finally, the sections were mounted using an anti-fade fluorescence mounting medium containing DAPI (P0131, Beyotime, China) and immediately observed and imaged under a fluorescence microscope. Each group included 6 animals, and one section from each animal was stained and analyzed. One field of view was selected for imaging. The images were analyzed using ImageJ 1.48 software (version 1.48, National Institutes of Health, USA) to calculate the percentage of the area with positive staining (Zhang et al. 2023).

### Western blot (WB)

Cells or tissue extracts were lysed using radio immunoprecipitation assay (RIPA) lysis buffer (P0013B, Beyotime, Shanghai, China) containing 1% PMSF (Phenylmethanesulfonyl fluoride) to extract total protein, following the manufacturer’s instructions. The supernatant was collected, and the total protein concentration for each sample was determined using the BCA Protein Assay Kit (P0011, Beyotime, Shanghai, China). Protein concentrations were adjusted to 1 µg/µL. Each sample (100 µL) was boiled at 100 °C for 10 min to denature the proteins and then stored at −80 °C until use. Based on the target protein’s molecular weight, 8–12% SDS-PAGE (Sodium dodecyl sulfate-polyacrylamide gel electrophoresis) gels were prepared. Using a micropipette, 50 µg of protein sample was loaded into each lane, and electrophoresis was performed at a constant voltage of 80 V initially, then increased to 120 V for 2 h to separate the proteins. The proteins were transferred from the gel to a PVDF membrane (1620177, Bio-Rad, USA) using a wet transfer method at a constant current of 250 mA for 90 min.

After protein transfer, the PVDF membrane was blocked with 5% non-fat milk in 1×TBST at room temperature for 1 h. The blocking solution was then removed, and the membrane was washed with 1×TBST for 10 min. The membrane was incubated overnight at 4 °C with primary antibodies (details in Table S2). After incubation, the membrane was washed three times with 1×TBST, each for 10 min. Next, the membrane was incubated at room temperature with HRP-conjugated secondary antibodies (goat anti-rabbit IgG, ab6721, Abcam, Cambridge, UK, dilution 1:5000; or goat anti-mouse IgG, ab205719, Abcam, Cambridge, UK, dilution 1:5000) for 1 h. The membrane was then washed three times with 1×TBST at room temperature, each for 5 min. The membrane was immersed in ECL reagent (1705062, Bio-Rad, USA) and incubated at room temperature for 1 min. Excess reagent was removed, and the membrane was covered with plastic wrap. The bands were visualized using the GE Image Quant LAS 4000 C imaging system. The relative expression levels of the target proteins were determined by normalizing the intensity of the target bands to the intensity of the β-actin bands. Each experiment was repeated three times (Chen et al. 2024).

### ELISA

The concentrations of IL-1β, IL-6, and TNF in rat serum samples were measured using ELISA kits. Blood samples were collected from the rats into tubes containing anticoagulants, and serum was separated by centrifugation at 3000 rpm for 10 min. The assays were conducted using commercial ELISA kits (IL-1β: RLB00-1, IL-6: R6000B, TNF: RTA00-1, R&D Systems, USA) following the manufacturer’s instructions. First, 200 µL of coating buffer containing the capture antibody was added to each well of a 96-well plate and incubated overnight at 4 °C. The next day, the coating solution was discarded, and each well was washed three times with 300 µL of wash buffer, each wash lasting 5 min. Then, 100 µL of blocking buffer was added to each well and incubated at room temperature for 2 h. After discarding the blocking buffer, 100 µL of diluted serum samples (1:2 dilution) and standards were added to each well and incubated for 2 h. The plate was washed four times with wash buffer, followed by the addition of 100 µL of diluted biotin-labeled detection antibody to each well and incubation for 1 h at room temperature. Subsequent steps included washing, incubation with streptavidin-HRP, substrate reaction, and absorbance measurement. The plate was washed four times with wash buffer, followed by the addition of 100 µL of diluted biotin-labeled detection antibody to each well and incubation for 1 h at room temperature. The plate was washed four times again, and 100 µL of HRP-conjugated streptavidin was added to each well and incubated at room temperature for 20 min. After another four washes, 100 µL of substrate solution was added to each well and incubated in the dark for 20 min until color development. Finally, 50 µL of stop solution was added to each well, and absorbance was read at 450 nm with background correction at 540 nm using a microplate reader (SpectraMax i3x, Molecular Devices, USA). The concentrations of IL-1β, IL-6, and TNF in the samples were calculated based on the standard curve. All samples were tested in triplicate, and data were presented as mean ± standard deviation. Statistical analysis was performed using GraphPad Prism 9 software (Huang et al. 2021).

### Statistical analysis

Data were obtained from at least three independent experiments and are presented as mean ± standard deviation (Mean ± SD). Differences between the two groups were compared using an independent samples t-test. A one-way analysis of variance (ANOVA) was used to compare three or more groups. If the ANOVA results indicated significant differences, Tukey’s HSD post hoc test was performed to compare differences between groups. For data that did not follow a normal distribution or had unequal variances, the Mann-Whitney U test or Kruskal-Wallis H test was used. All statistical analyses were conducted using GraphPad Prism 9 and R software. A significance level of 0.05 was set, and two-sided *p*-values less than 0.05 were considered statistically significant (Taghizade et al. 2024).

## Results

### GSEA combined with immune infiltration reveals involvement of AP in inflammatory response

In investigating the gene expression changes associated with AP, we first downloaded the transcriptome dataset GSE194331 from the GEO database.

**Fig. 1:**
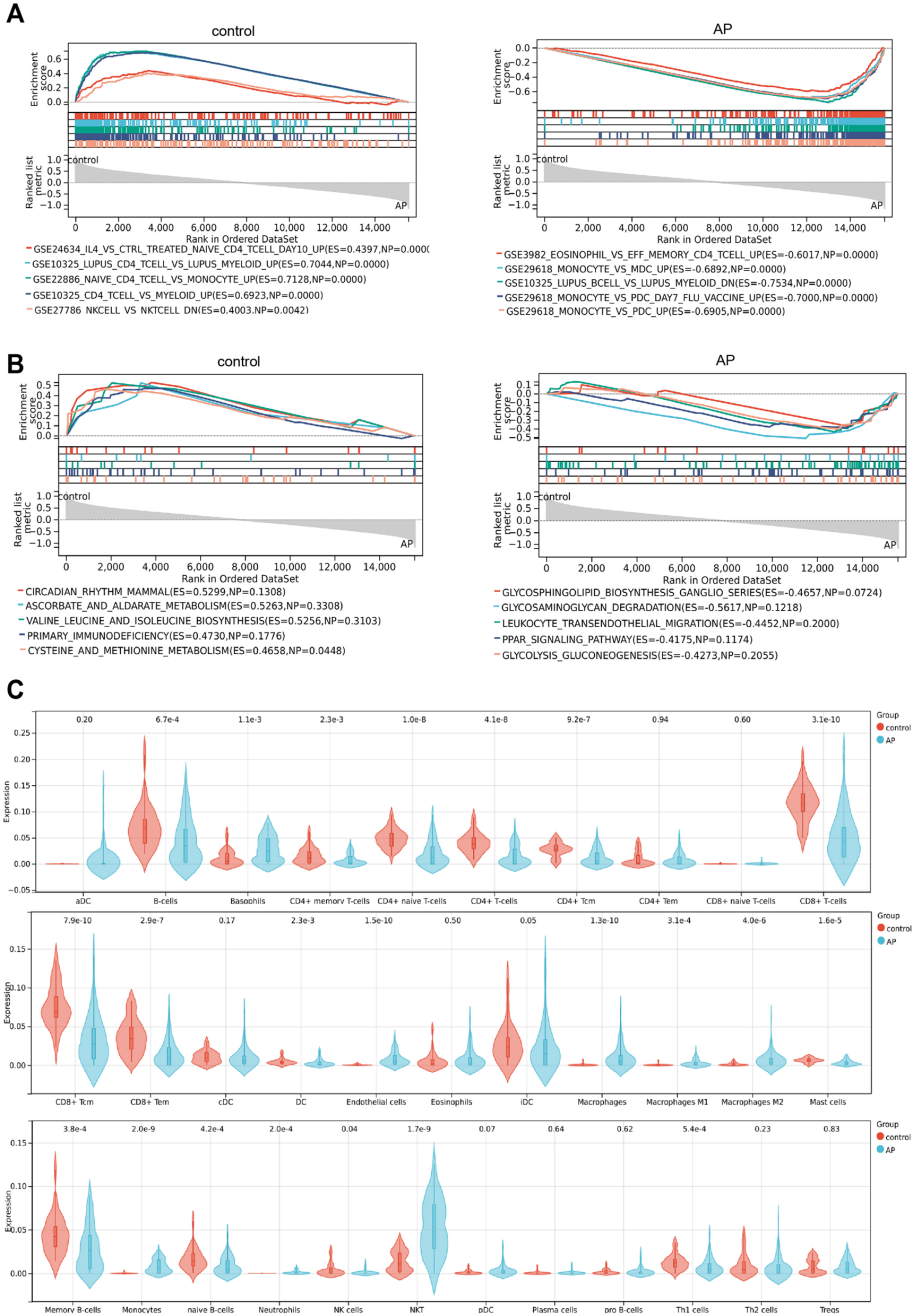
Immune Mechanisms and Enriched Signaling Pathways in AP. Note: **A** GSEA showing immune gene sets enriched in the control group and the AP group; **B** KEGG gene sets enriched in the control group and the AP group; **C** Violin plot of xCell immune infiltration analysis results, AP: *n* = 87, control: *n* = 32

GSEA enrichment analysis was performed using ImmuneSigDB gene sets. The results showed that the control group was primarily enriched in gene sets related to CD4^+^ T cells and natural killer (NK) cells. In contrast, the AP group was mainly enriched in gene sets related to basophils, monocytes, and B cells (Fig. 1A).

Next, GSEA enrichment analysis was performed using KEGG gene sets. The control group was primarily enriched in gene sets related to mammalian circadian rhythm, ascorbate and alternate metabolism, branched-chain amino acid biosynthesis pathways (such as valine, leucine, and isoleucine), sulfur amino acid metabolism pathways (including cysteine and methionine), and genes associated with primary immunodeficiency. In contrast, the AP group was mainly enriched in gene sets related to mannose-type O-glycan biosynthesis, glycosaminoglycan degradation, leukocyte transendothelial migration, peroxisome proliferator-activated receptor (PPAR) signaling pathway, as well as glycolysis and gluconeogenesis pathways (Fig. 1B).

Using the xCell immune infiltration method to analyze the immune microenvironment in the AP-related dataset, we found significant changes in various immune cells, including CD4^+^ T cells, CD8^+^ T cells, and macrophages (Fig. 1C).

By systematically analyzing the gene expression patterns associated with AP, this study reveals the dynamic changes in different immune cell populations and the regulation of related metabolic pathways in AP.

### Screening of key genes involved in AP

The aforementioned findings revealed significant changes in various immune cells in the peripheral blood of AP patients. KEGG analysis indicated that multiple glucose metabolism pathways, including the glycolysis signaling pathway, were highly enriched in the AP group.

**Fig. 2:**
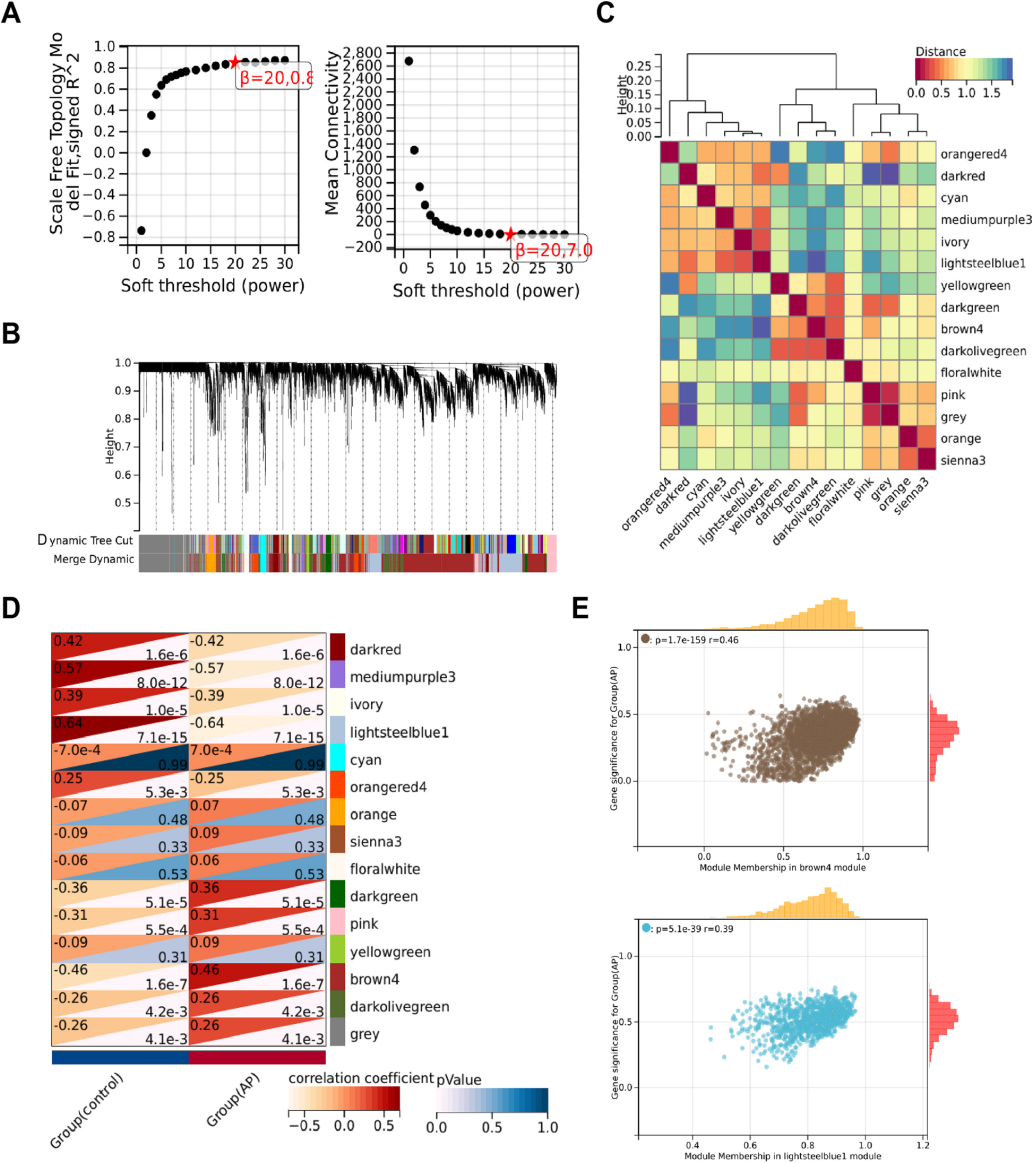
WGCNA Analysis of AP Transcriptomic Data. Note: **A** Scale independence, mean connectivity, and the scale-free topology fit index plot, determining the weighted value β = 20 that satisfies the scale-free network law; **B** Clustering dendrogram of co-expression network modules; **C** Correlation heatmap among the 15 modules; **D** Correlation analysis between modules and AP traits; **E** Scatter plots of module-trait relationships for brown4 and lightsteelblue1 modules with AP

Through WGCNA, gene modules closely related to the pathological state of AP were further screened. A soft-threshold power of β = 20 (R^2^ = 0.8) was selected to establish a scale-free network (Fig. 2A). A clustering dendrogram identified 15 modules (Fig. 2B), and the correlations of these 15 modules are shown in Fig. 2C.

Further identification of modules significantly associated with AP revealed that the brown4 module had a significant positive correlation with AP (cor = 0.46, *P* = 1.6e-7), while the lightsteelblue1 module had a significant negative correlation with AP (cor = −0.64, *P* = 7.1e-15) (Fig. 2D). Scatter plots further confirmed the significant association of the brown4 and lightsteelblue1 modules with the AP phenotype (Fig. 2E). Therefore, we selected the genes from these two modules, totaling 1817 genes, as significantly associated with AP. The expression of these 1817 genes may reflect changes in immune responses and the regulation of metabolic pathways related to the pathological state of AP.

**Fig. 3:**
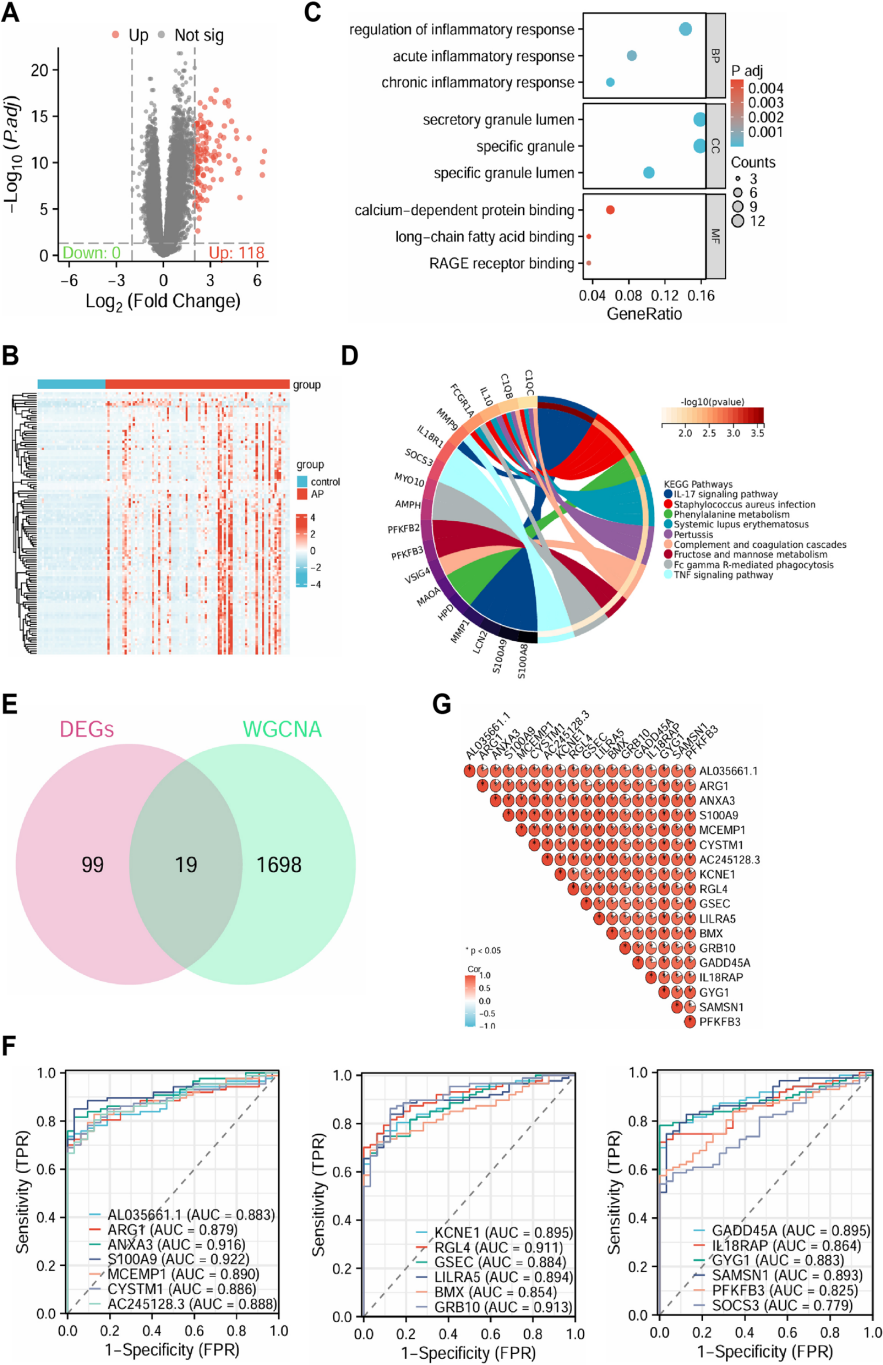
Differential Analysis of Bulk RNA-seq Data. Note: **A** Volcano plot of differential analysis, red dots represent significantly upregulated genes, gray dots represent non-significant genes, AP: *n* = 87, control: *n* = 32; **B** Heatmap of DEGs; **C**-**D** GO (**C**) and KEGG (**D**) enrichment analysis of DEGs; **E** Venn diagram showing the overlap between DEGs and WGCNA-identified AP-associated genes; **F** ROC diagnostic analysis of the 19 intersecting genes, with the X-axis representing 1-specificity (false positive rate, closer to zero indicates higher accuracy) and the Y-axis representing sensitivity (true positive rate, higher values indicate better accuracy); **G** Correlation analysis of 18 genes, with the central pie chart indicating *p*-values from correlation tests, * denotes *p*-value < 0.05. AP: *n* = 87, control: *n* = 32

Through differential expression analysis, 118 significant DEGs were identified, all of which were significantly upregulated (Fig. 3A-B). Enrichment analysis of these genes revealed the following: GO enrichment analysis showed that, in terms of biological processes (BP), the DEGs were significantly associated with the regulation of inflammatory response, acute inflammatory response, and chronic inflammatory response. In terms of cellular components (CC), the DEGs were significantly associated with the secretory granule lumen, specific granule, and specific granule lumen. For molecular functions (MF), the DEGs were significantly associated with calcium-dependent protein binding, long-chain fatty acid binding, and RAGE receptor binding (Fig. 3C). KEGG pathway analysis indicated that the DEGs were primarily enriched in the IL-17 signaling pathway, Fructose and mannose metabolism, and TNF signaling pathway (Fig. 3D).

DEGs and the AP-related genes identified through WGCNA were intersected, resulting in 19 genes (Fig. 3E). ROC curve analysis demonstrated the diagnostic performance of these 19 genes, showing that except for SOCS3, the AUC values of the other 18 genes were all greater than 0.8, indicating high diagnostic accuracy (Fig. 3F). Correlation analysis of these 18 genes, after excluding SOCS3, revealed significant positive correlations among them (Fig. 3G). Therefore, we defined these 18 genes as characteristic genes of AP.

Through the integration of WGCNA and DEG analysis, this study provides an in-depth molecular understanding of the pathological mechanisms of AP, identifying gene modules significantly associated with AP and revealing key pathways related to inflammation and metabolism.

### Single-cell analysis reveals PFKFB3 as a key regulator in AP

A series of in-depth gene expression analyses revealed the key molecular mechanisms and diagnostic markers of AP, paving the way for further exploration of cellular heterogeneity and the potential molecular basis of AP using single-cell technologies. We downloaded the scRNA-seq data GSE250486 for AP mice from public databases and integrated the data using the Seurat package. Most cells had nFeature_RNA < 5000, nCount_RNA < 20,000, and percent.mt < 20% (Figure S1A). After removing low-quality cells based on these criteria, we obtained an expression matrix of 17,876 genes and 19,690 cells. Correlation analysis of sequencing depth showed that in the filtered data, the correlation coefficient between nCount_RNA and percent.mt was *r* = −0.13, and the correlation coefficient between nCount_RNA and nFeature_RNA was *r* = 0.68 (Figure S1B). This indicates that the filtered cell data was of good quality and suitable for further analysis.

Further analysis was conducted on the filtered cells, followed by initial normalization of the data. Based on the selected highly variable genes, linear dimensionality reduction was performed using PCA (Figure S1C), showing the distribution of cells on PC_1 and PC_2 (Figure S1D). The results indicated the presence of batch effects among the samples.

We used the Harmony package to correct batch effects in the sample data for subsequent analysis (Figure S1E). The standard deviation of the PCs was sorted using an ElbowPlot (Figure S1F). The corrected results showed that the batch effects were effectively eliminated (Figure S2A). Next, the UMAP algorithm was applied for nonlinear dimensionality reduction based on the top 20 PCss, and clustering at different resolutions was visualized using the Clustree package (Figure S2B). Through UMAP clustering analysis, we divided all the cells into 35 clusters (Figure S2C-D).

**Fig. 4:**
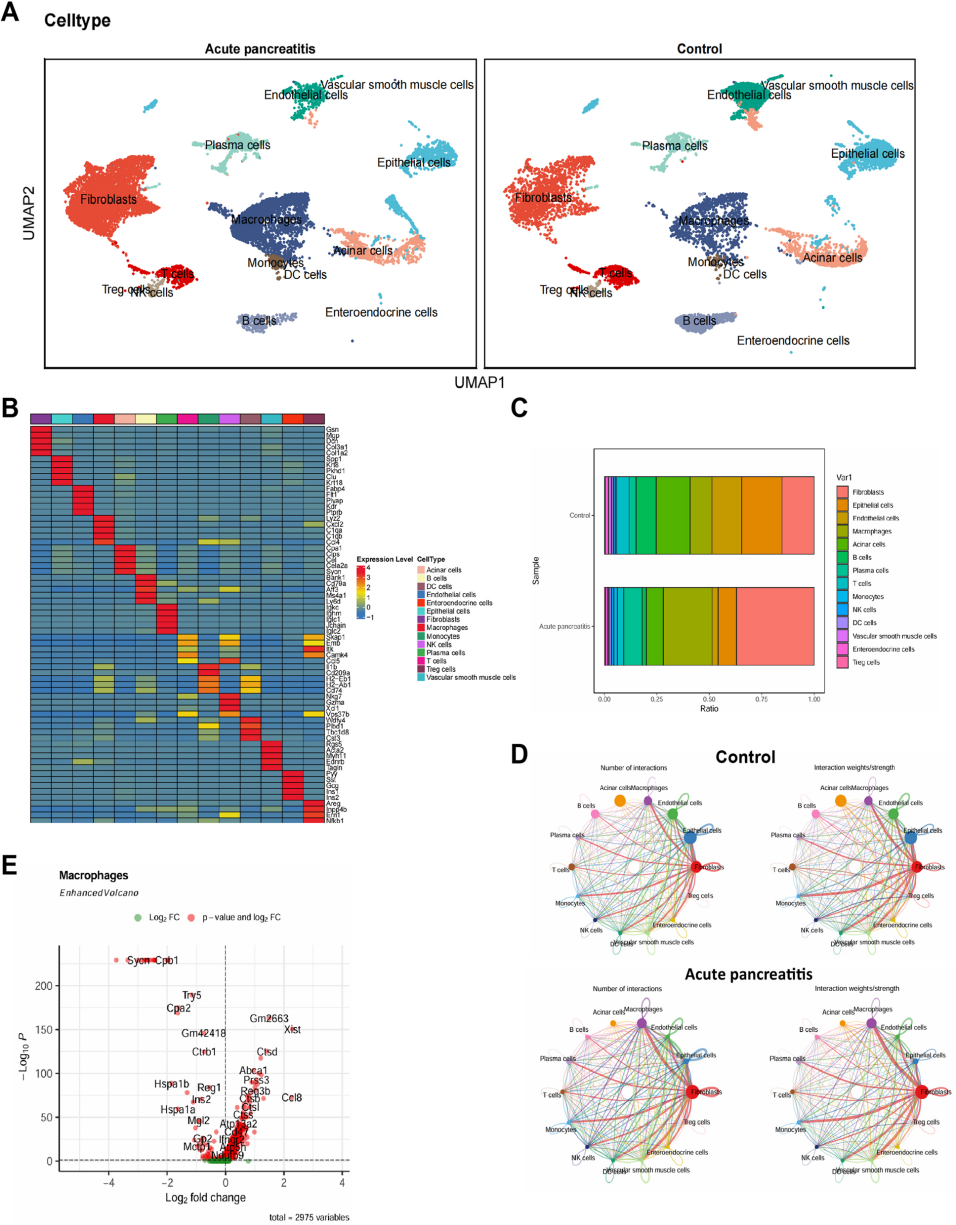
Cellular Differences in AP-associated scRNA-seq. Note: **A** UMAP-based visualization of cell annotation results by group; **B** Heatmap of the top 5 gene expression correlations in 14 cell types; **C** Proportional representation of different cell subpopulations in each sample, with cell types indicated by different colors; **D** Circos plot of cell communication in samples, with line thickness in the left plot indicating the number of pathways and in the right plot indicating interaction strength; **E** Volcano plot of differential analysis for two groups of macrophages. AP: *n* = 2, control: *n* = 2

Then, we used the Bioconductor/R package “SingleR” for automatic annotation of the 35 cell clusters, identifying 14 cell types (Fig. 4A). The reliability of the clustering was validated using an expression heatmap (Fig. 4B). Additionally, the proportion of each cell type in each group was shown, revealing that the proportion of macrophages increased most significantly in the AP group (Fig. 4C).

Next, we used the “CellChat” package to explore the communication pathways between different cell types, particularly noting a significant increase in interactions between macrophages and other cell types in the AP group (Fig. 4D). Therefore, we selected macrophages for differential analysis, ultimately identifying 2975 DEGs (Fig. 4E).

Additionally, intersecting the DEGs in scRNA-seq macrophages with the 18 characteristic genes from bulk RNA-seq identified three overlapping genes: ANXA3, PFKFB3, and SAMSN1 (Figure S3A). The expression profiles of these three genes in the scRNA-seq dataset showed that ANXA3 was widely expressed, while PFKFB3 and SAMSN1 were primarily expressed in macrophages and T cells (Figure S3B).

The expression differences of the three genes in macrophages between the two groups were also shown, indicating that PFKFB3 exhibited higher differential expression compared to SAMSN1, with significantly higher expression in macrophages in the AP group (Figure S3C). Furthermore, integrating the bulk RNA-seq data, PFKFB3 was significantly enriched in the Fructose and mannose metabolism signaling pathway. Studies have shown that PFKFB3 can drive the glycolysis pathway (Zeng et al. 2022; De Bock et al. 2013). Elevated PFKFB3 is closely associated with excessive inflammatory responses and high mortality in sepsis (Xiao et al. 2023). The PFKFB3 inhibitor KAN0438757 has been shown to prevent the progression of severe AP (Ergashev et al. 2024).

By integrating scRNA-seq and bulk RNA-seq data analysis, we thoroughly investigated the dynamic changes in cell types and intercellular communication mechanisms in the AP mouse model. In particular, we focused on the role of the PFKFB3 gene in the glycolysis pathway and its potential involvement in the excessive inflammatory response associated with AP.

### Delivery of PFKFB3 inhibitor using nanovesicle carriers

**Fig. 5:**
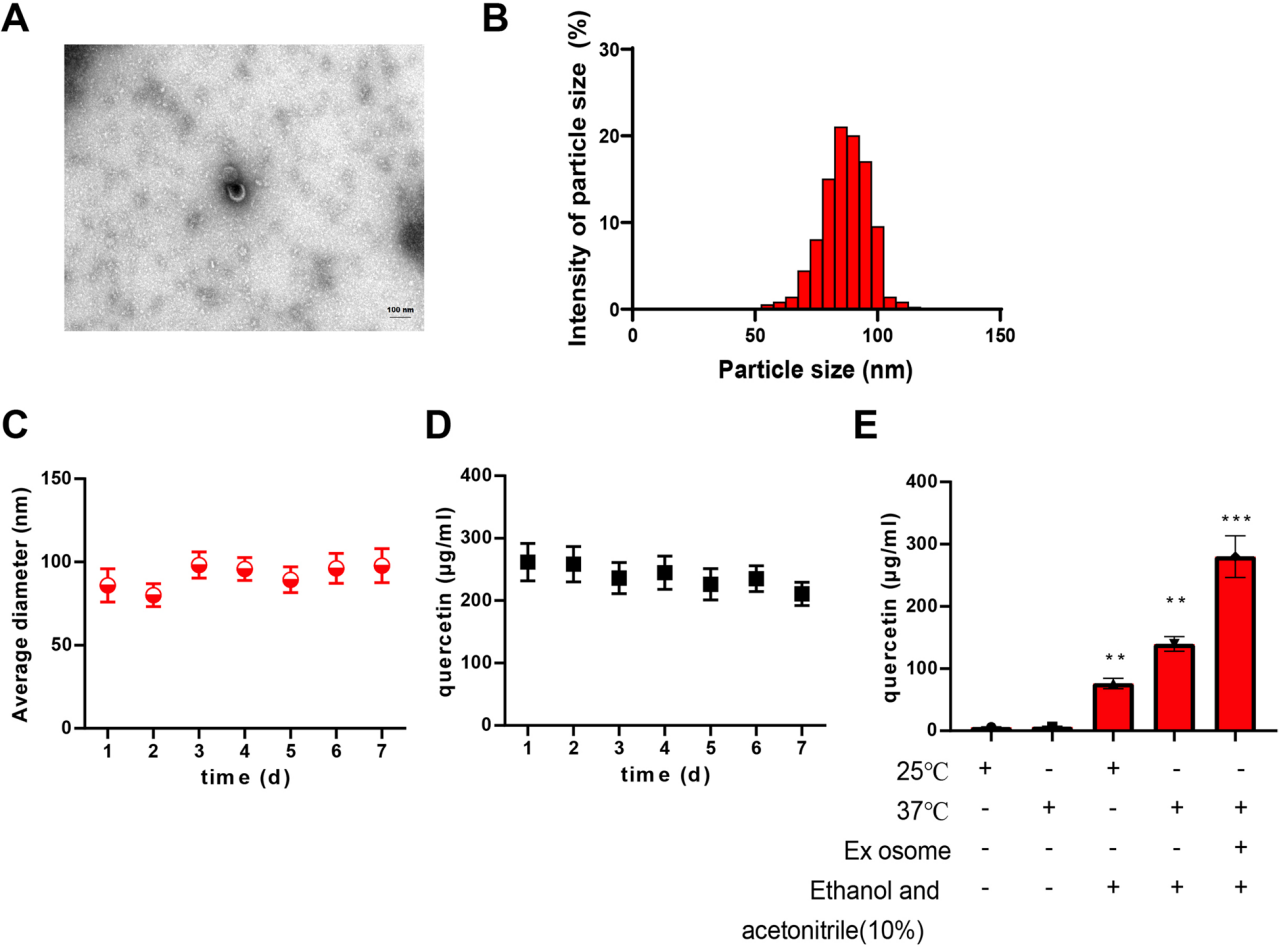
Characterization and Functionalization of Q-Ex. Note: **A** TEM image of EVs (×70,000), scale bar = 100 nm; **B** Particle size distribution of Q-Ex; **C** Size variation of Q-Ex stored in serum at 37 °C; **D** Concentration variation of quercetin in Q-Ex stored in serum at 37 °C; **E** Solubility of quercetin under different conditions detected by HPLC. Compared with the 25 °C group, *p < 0.05, **p < 0.01, ***p < 0.001. Cell experiments were repeated three times

In modern drug delivery systems, nanovesicles have gained attention as an emerging carrier due to their unique biocompatibility and tunability. Nanovesicles can effectively enhance drug stability and bioavailability, thereby improving therapeutic efficacy. In this study, we selected nanovesicles as carriers to achieve efficient delivery of the PFKFB3 inhibitor. Nanovesicles from AR42 J cell membranes were successfully isolated using differential centrifugation. TEM revealed their uniformity, with a diameter of approximately 85 nm (Fig. 5A). The size distribution of Q-Ex was shown to be 86.2 ± 6.5 nm, consistent with the TEM results (Fig. 5B). To determine the stability of Q-Ex in vitro, we further studied the size distribution of Q-Ex in serum over one week, and the results indicated that the average diameter remained stable (Fig. 5C). Additionally, we monitored the concentration of quercetin over a period of 7 days, and the results showed that its concentration remained stable, suggesting that the lipid bilayer of EVs provided a protective barrier (Fig. 5D). To enhance the solubility of Q-Ex and ensure its therapeutic efficacy, we established an HPLC method to determine the solubility of quercetin under different conditions. The addition of ethanol and acetonitrile (10%) or increasing the temperature to 37 °C significantly improved the solubility of quercetin. This difference was especially notable compared to the solubility of free quercetin at 25 °C without ethanol and acetonitrile, indicating that Q-Ex has higher solubility than free quercetin (Fig. 5E).

These results indicate that we successfully loaded the PFKFB3 small molecule inhibitor quercetin into EVs, demonstrating stability and good solubility. This lays the foundation for further research on its therapeutic effects in vitro and in vivo.

### Inhibition of LPS-induced pro-inflammatory cytokine release and apoptosis in macrophages by Q-Ex in vitro

**Fig. 6:**
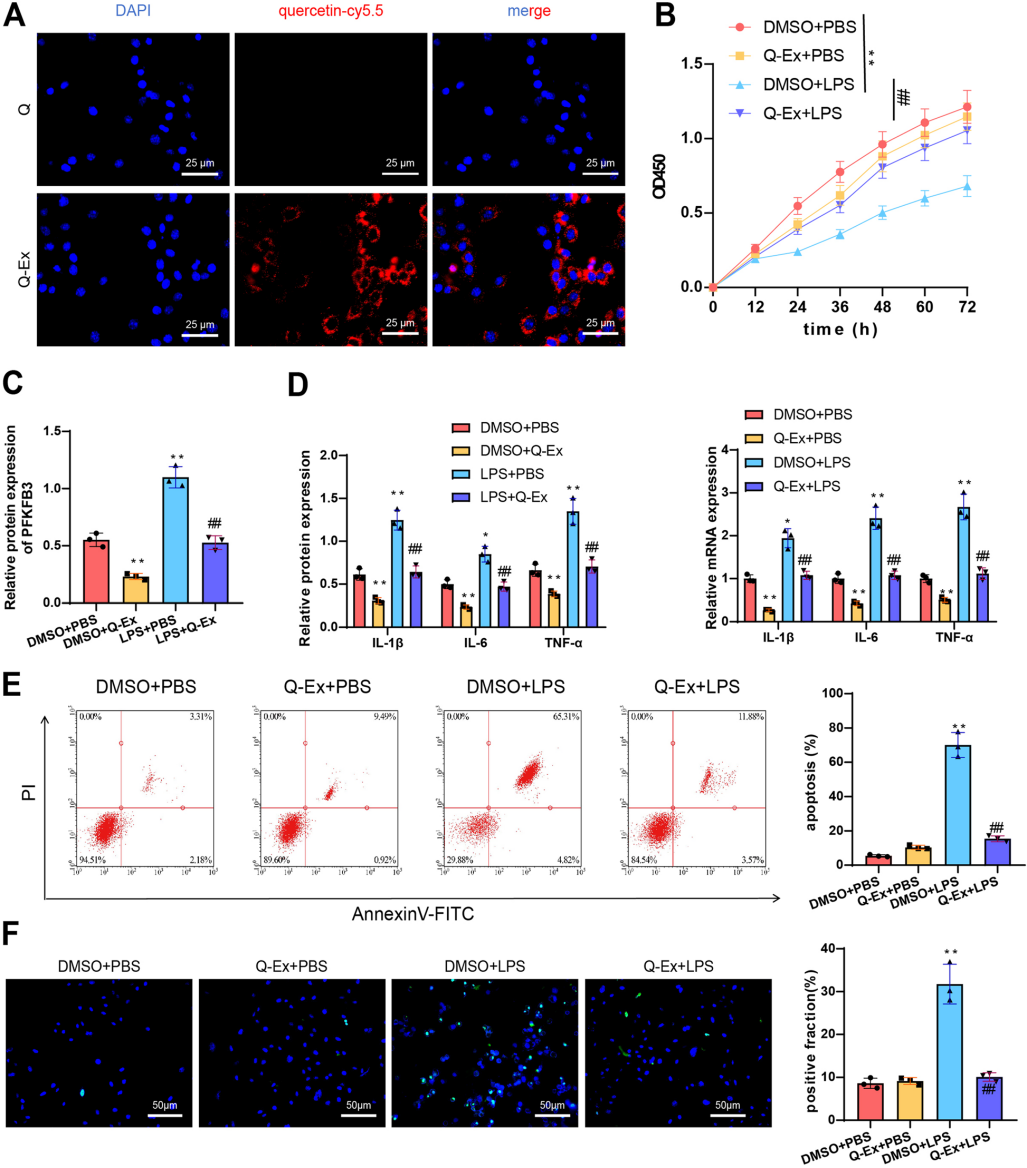
Cellular Uptake of Q-Ex and Its Anti-inflammatory and Anti-apoptotic Effects in vitro Note: **A** Fluorescence microscopy images showing the targeting ability of Q-Ex and quercetin (Q) in vitro using Cy5.5-labeled quercetin (×400), bar = 25 μm; **B** Cell viability assay (CCK-8) of different treatment groups; **C** WB analysis of PFKFB3 protein expression in various treatment groups; **D** WB and RT-qPCR analysis of pro-inflammatory cytokine protein levels in different groups; **E** Flow cytometry analysis of apoptosis levels and bar chart of apoptotic cell percentages in different groups; **F** TUNEL staining to observe apoptosis levels and bar chart of positive cell percentages, (×200)scale bar = 50 μm. Cell experiments were repeated three times. Compared with the DMSO + PBS group, *p < 0.05, **p < 0.01; compared with the DMSO + LPS group, #*p* < 0.05, ##*p* < 0.01

To assess cellular uptake, we labeled quercetin with Cy5.5. Fluorescence microscopy revealed that Q-Ex enhanced the cellular uptake of quercetin (Fig. 6A). To simulate an inflammatory response in vitro, we induced inflammation in RAW264.7 macrophages using LPS. A DMSO + PBS group was established as a control, and a Q-Ex + PBS group was included to observe if Q-Ex directly affected the cells. Cell viability was first assessed using the CCK-8 assay. The results showed that Q-Ex did not directly inhibit cell viability, whereas LPS treatment significantly reduced cell viability. In the Q-Ex + LPS group, cell viability was restored to levels similar to the control group, suggesting a protective effect of Q-Ex on the cells (Fig. 6B).

Next, we examined the expression of PFKFB3 protein in the different groups of cells using WB. The results showed that Q-Ex reduced PFKFB3 protein levels in RAW264.7 cells. In the LPS-induced group and the DMSO + LPS group, PFKFB3 expression was upregulated, whereas Q-Ex was able to reverse this upregulation (Fig. 6C). Regarding the expression of pro-inflammatory cytokines TNF-α, IL-1β, and IL-6, Western blot and RT-qPCR results indicated that Q-Ex significantly inhibited their expression. Compared to the DMSO + PBS group, the expression levels of pro-inflammatory cytokines were significantly lower in the Q-Ex + PBS group. In contrast, LPS induction markedly increased the expression of these cytokines, and Q-Ex exhibited an inhibitory effect. The PCR results were consistent with the findings from the WB analysis (Fig. 6D).

Furthermore, we assessed cell apoptosis using flow cytometry. The results showed no significant difference between the Q-Ex + PBS group and the DMSO + PBS group, indicating that Q-Ex does not directly induce cell apoptosis. Under LPS induction, the level of apoptosis significantly increased; however, Q-Ex treatment reduced this apoptosis level (Fig. 6E). TUNEL staining results were consistent with the flow cytometry findings. Specifically, no TUNEL-positive staining was observed in the Q-Ex + PBS group, whereas LPS treatment significantly increased the number of TUNEL-positive nuclei, with the percentage of positive cells reaching 45%. However, Q-Ex treatment significantly reduced the level of apoptosis (Fig. 6F).

In summary, these results demonstrate that Q-Ex is effectively taken up by cells in vitro and can inhibit LPS-induced pro-inflammatory cytokine secretion and cell apoptosis in RAW264.7 cells.

### Q-Ex inhibits LPS-induced changes in glucose metabolism and oxidative stress in macrophages in vitro

**Fig. 7:**
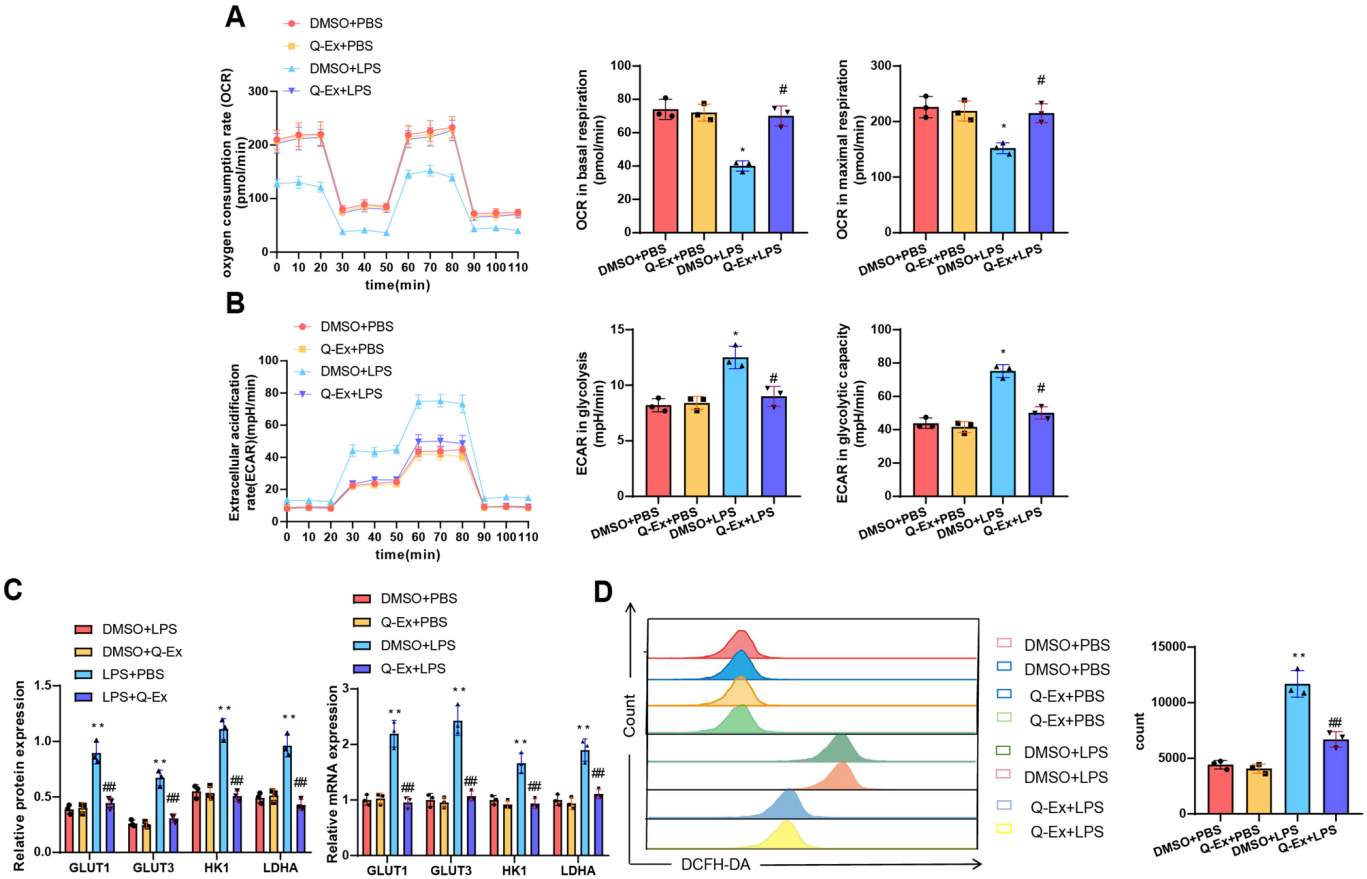
Effects of Q-Ex on Glucose Metabolism and Oxidative Stress in RAW264.7 Cells. Note: **A** Measurement of the OCR in cells treated with quercetin using Seahorse XF Analyzer; **B** Measurement of the ECAR in cells treated with quercetin using Seahorse XF Analyzer; **C** WB and RT-qPCR analysis of key glycolysis enzyme protein levels in various treatment groups; **D** Flow cytometry analysis of DCFH-DA positive cells and bar chart of the number of positive cells in different groups. Cell experiments were repeated three times. Compared with the DMSO + PBS group, *p < 0.05, **p < 0.01, ***p < 0.001; compared with the DMSO + LPS group, #*p* < 0.05, ##*p* < 0.01

PFKFB3 is an enzyme that plays a crucial role in glucose metabolism, particularly in regulating the rate of glycolysis (Pang et al. 2024). Inhibiting PFKFB3 not only affects the glycolysis pathway but may also have significant impacts on the overall energy metabolism of cells (Imbert-Fernandez et al. 2024). To explore the specific effects of Q-Ex on PFKFB3 inhibition, we measured the OCR and ECAR of the cells in each group using the Seahorse XF analyzer. The results showed that LPS treatment significantly reduced OCR, while Q-Ex treatment significantly increased OCR. Additionally, LPS increased ECAR, which was inhibited by Q-Ex, indicating that Q-Ex can significantly suppress cellular energy metabolism activities (Fig. 7A-B). WB and RT-qPCR analysis of key glycolysis enzymes showed that LPS increased the expression levels of GLUT1, GLUT3, HK1, and LDHA, which were inhibited by Q-Ex (Fig. 7C). To assess the ROS scavenging ability of Q-Ex, cells in each group were labeled with DCFH-DA and the positive cells were detected using flow cytometry (Abdal Dayem et al. 2025). The results indicated that LPS induced a significant release of ROS in RAW264.7 cells, which was inhibited by Q-Ex, suggesting an inhibitory effect of Q-Ex on oxidative stress (Fig. 7D).

These findings indicate that Q-Ex can inhibit LPS-induced increases in glycolysis rate and oxidative stress response.

### Q-Ex significantly reduces inflammation and improves metabolic state in pancreatitis rats

In the previous studies, we observed the protective effects of Q-Ex on LPS-induced inflammation in macrophages in vitro. To further explore the in vivo effects of Q-Ex, we established a rat AP model and used DMSO + NS as a control group while also setting up a Q-Ex + NS group to observe the effects of Q-Ex in vivo.

**Fig. 8:**
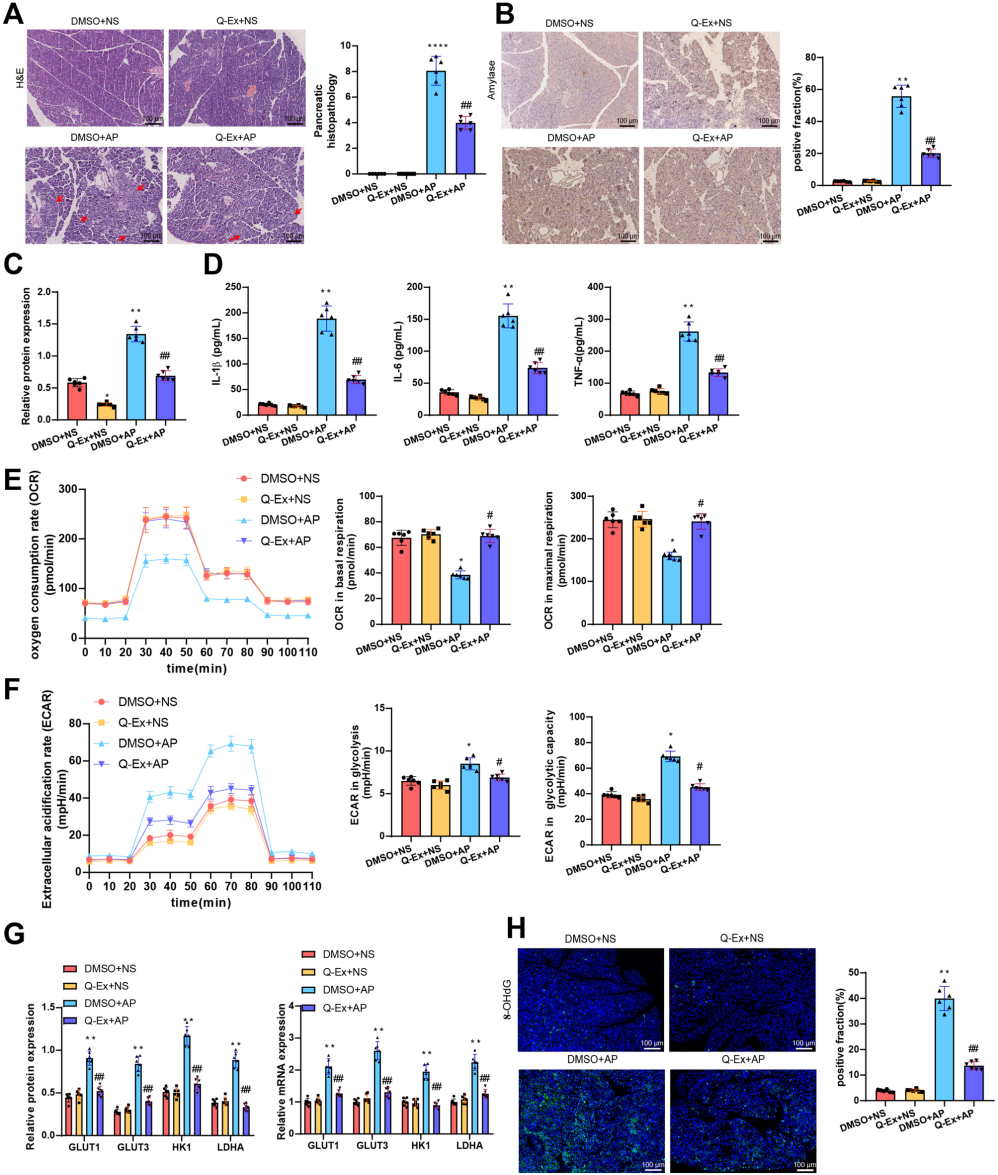
Anti-inflammatory and Metabolic Improvement Effects of Q-Ex in a Rat Model of AP. Note: **A** H&E staining of pancreatic tissue showing pathological changes and histopathological scores, scale bar = 200 μm; **B** Immunohistochemical staining of pancreatic tissue sections showing amylase-positive levels and the percentage of positive areas, scale bar = 200 μm; **C** WB analysis of PFKFB3 expression levels and grayscale value statistics in pancreatic tissue; **D** ELISA detection of pro-inflammatory cytokine levels in rat serum; **E** Measurement of glycolysis rate and OCR in pancreatic tissue using Seahorse XF Analyzer; **F** Measurement of ECAR in pancreatic tissue using Seahorse XF Analyzer; **G** WB and RT-qPCR analysis of key glycolysis enzyme expression levels and grayscale value statistics in pancreatic tissue; **H** Immunofluorescence staining of pancreatic tissue sections showing 8-OHdG (DNA oxidative damage marker) levels and percentage of positive staining, scale bar = 100 μm. Each group consisted of 6 rats. Compared with the DMSO + NS group, *p < 0.05, **p < 0.01, ***p < 0.001, ****p < 0.0001; compared with the DMSO + AP group, #*p* < 0.05, ##*p* < 0.01, ###*p* < 0.001

First, we examined the morphology of pancreatic tissues from each group using H&E staining. The results showed that in the cerulein-induced AP model, there were evident signs of pancreatic edema, release of inflammatory factors, and partial tissue necrosis. However, these pathological changes were alleviated after Q-Ex treatment. The pathological scoring results indicated that Q-Ex did not directly cause pancreatic lesions but significantly reduced cerulein-induced AP lesions (Fig. 8A). Additionally, immunohistochemical staining for amylase expression revealed strong positive expression in the cerulein-induced AP model, which significantly decreased after Q-Ex treatment (Fig. 8B).

Next, we examined the expression levels of PFKFB3 in pancreatic tissues from each group of rat models using WB analysis. The results showed that Q-Ex significantly inhibited the expression of PFKFB3, both in control rats and in the AP model rats (Fig. 8C). To further assess the inflammatory response, we measured the levels of TNF-α, IL-1β, and IL-6 in the serum of each group of rats using ELISA. The results indicated that the levels of these pro-inflammatory cytokines were significantly increased in the AP model rats, whereas these levels decreased following Q-Ex treatment (Fig. 8D).

In previous studies, we have demonstrated the impact of Q-Ex on the metabolic levels of macrophages in vitro. To further validate our findings in vivo, we measured the glycolysis rate in the pancreatic tissues of rats. We used the Seahorse XF analyzer to measure the OCR and ECAR). The results showed that OCR was significantly decreased in the pancreatic tissues of rats in the AP model group. However, OCR significantly increased following Q-Ex treatment. Conversely, ECAR was elevated in the AP model rats and was inhibited by Q-Ex, indicating that Q-Ex could significantly suppress cellular energy metabolism activities (Fig. 8E-F). Additionally, we assessed the expression of key glycolysis enzymes using WB and RT-qPCR. The results indicated that the expression levels of GLUT1, GLUT3, HK1, and LDHA were significantly upregulated in the pancreatic tissues of AP model rats, and this upregulation was inhibited by Q-Ex (Fig. 8G). For oxidative stress levels, we performed immunofluorescence staining for the DNA oxidative damage marker 8-OHdG. The results showed strong positive fluorescence in the pancreatic tissue sections of rats in the DMSO + AP group, indicating a high level of oxidative stress. Under Q-Ex treatment, the positive fluorescence significantly decreased, demonstrating the ability of Q-Ex to inhibit oxidative stress (Fig. 8H).

Overall, the results of this study indicate that EVs-delivered PFKFB3 small molecule inhibitor quercetin can not only inhibit the inflammatory response in AP but also potentially improve glucose metabolism disorders by regulating glycolysis-related pathways and inhibiting oxidative stress. This provides a new therapeutic strategy for the treatment of AP.

## Discussion

Acute pancreatitis (AP) is a common acute disease of the digestive system, characterized by acute pancreatic inflammation and metabolic disorders (Szatmary et al. 2022; Zerem et al. 2023; Forman et al. 2021). Previous studies have confirmed that abnormalities in glucose metabolism play a crucial role in the pathological process of AP. However, the specific molecular mechanisms remain unclear (Whitcomb 2022). This study explores the role of PFKFB3 in glucose metabolism disorder following AP through bioinformatics analysis and experimental validation, providing new perspectives and strategies for AP treatment.

Given the critical role of glucose metabolism disorders in AP, we focused on PFKFB3, a key glycolytic regulatory enzyme whose activity is closely linked to various metabolic and inflammatory diseases (Xie et al. 2025; Zeng et al. 2022; Min et al. 2021; Kasprzak 2021). Previous research has primarily focused on the expression of PFKFB3 and its role in metabolic regulation, with limited attention to its specific function in AP (Xiao et al. 2023). This study systematically identified significant changes in PFKFB3 in AP by analyzing transcriptome data from the GEO database, combined with GSEA and WGCNA methods. These findings were consistent with previous reports (Zhang et al. 2024a, b), and further validated its key role in glucose metabolism and inflammatory responses.

Based on our understanding of PFKFB3’s role in AP, we further explored the application of cell membrane nanovesicles as a novel drug delivery platform for AP treatment. Nanovesicles exhibit superior biocompatibility and targeting ability compared to conventional delivery systems. They can more effectively preserve drug activity, enhance stability in vivo, and promote targeted delivery (Ding et al. 2025; Ou et al. 2023; Feng et al. 2022; Wang et al. 2023a, b). In this study, we loaded the PFKFB3 small molecule inhibitor quercetin into cell membrane nanovesicles for the first time and validated its potential in AP treatment through a series of in vitro and in vivo experiments. The results showed that Q-Ex significantly reduced the expression of PFKFB3, decreased the release of pro-inflammatory cytokines. These findings align with previous studies reporting the enhanced anti-inflammatory properties of quercetin-loaded EVs (Peng et al. 2024), ultimately inhibiting glycolysis and thereby improved glucose metabolism disorder.

To evaluate the therapeutic efficacy of Q-Ex in AP, we established an LPS-induced inflammatory model using RAW264.7 cells and a rat model of acute pancreatitis. Results from both models consistently supported the therapeutic potential of targeting PFKFB3 in AP (Zhang et al. 2024a, b; Li et al. 2023; Wang et al. 2022; Xu et al. 2021). In vitro experiments showed that Q-Ex significantly reduced the inflammatory response, enhanced macrophage activity, and inhibited apoptosis. This aligns with previous findings on the anti-inflammatory effects of quercetin(Kizilkan et al. 2025; Xiang et al. 2025), but this study further elucidates the mechanism by which quercetin achieves these effects through the regulation of PFKFB3. In vivo, experiments similarly demonstrated that tail vein injection of Q-Ex significantly reduced inflammatory markers in rat serum and markedly improved the pathological condition of pancreatic tissues. This validates the efficacy of PFKFB3 small molecule inhibitors loaded in nanovesicles in vivo.

Moreover, bioinformatics analysis played a pivotal role in this study. Systematic transcriptomic analysis revealed significantly altered metabolic and immune pathways in AP. Using GSEA and WGCNA, we identified gene modules strongly associated with AP pathology, particularly PFKFB3 (Xu et al. 2024; Wang et al. 2023a, b; Chen et al. 2022; Guo et al. 2022). Cell communication analysis further revealed the key role of macrophages in AP and their interactions with other cell types, providing new insights into the pathological mechanisms of AP.

Collectively, this study demonstrates that PFKFB3 small molecule inhibitors loaded in cell membrane nanovesicles can effectively improve glucose metabolism disorder and inflammatory response in an AP rat model. The integrated model of key DEGs screened by machine learning techniques improved diagnostic accuracy, while the nanovesicle delivery system ensured drug efficacy and target specificity. The experimental results showed significant improvements in inflammatory marker levels, glucose metabolism parameters, and pancreatic tissue pathology in treated model animals.

This study reveals the critical role of PFKFB3 in glucose metabolism disorder following AP and is the first to apply cell membrane nanovesicle technology to deliver the PFKFB3 small molecule inhibitor quercetin. The efficacy of this approach in improving glucose metabolism disorder and inflammatory response after AP was validated. Through systematic bioinformatics analysis and experimental validation, this study not only expands the understanding of the pathological mechanisms of AP but also provides a novel therapeutic strategy. This innovative approach holds potential clinical applications and may offer more effective treatment options for AP patients, improving their prognosis and quality of life.

Despite the promising outcomes, this study has certain limitations in terms of mechanistic exploration and translational potential. First, the results are primarily based on animal models and in vitro experiments and have not yet been validated in clinical trials. Second, the preparation and purification process of cell membrane nanovesicles is complex and costly, which may limit their large-scale application in clinical practice. Additionally, the limited sample size in the study may affect the generalizability and reliability of the results. Therefore, future research should focus on increasing the sample size and validating the findings in clinical trials.

To address these limitations, future studies should aim to optimize the preparation process of cell membrane nanovesicles to enhance production efficiency and stability while reducing costs, thereby promoting clinical application. Further research should also explore the effects of PFKFB3 small molecule inhibitors in different types of AP patients, verifying their safety and efficacy in clinical settings. Additionally, combining other potential therapeutic targets and strategies could be considered to develop comprehensive treatment plans aiming to provide more effective and holistic approaches for AP treatment. Through continued in-depth research, it is hoped that more practical and efficacious treatment options for AP patients can be developed.

## Conclusion

The use of PFKFB3 inhibitors delivered through cell membrane nanovesicles offers a promising new approach to treating acute pancreatitis (AP). By improving glucose metabolism and reducing inflammation, this method not only addresses key metabolic issues but also provides a foundation for future therapeutic developments. The findings support the potential of nanovesicle-based drug delivery systems, which could revolutionize treatment strategies for pancreatitis and other related disorders(Graphical Abstract). This innovative approach may lead to more effective management options, paving the way for improved patient outcomes in the treatment of AP.

## Supplementary Information

Supplementary Material 1. Figure S1. Quality Control, Filtering, and PCA of scRNA-seq Data. Note: (A) Violin plots of the number of genes per cell (nFeature_RNA), the number of mRNA molecules per cell (nCount_RNA), and the percentage of mitochondrial genes (percent.mt) in the scRNA-seq data; (B) Scatter plots showing correlations between nCount_RNA and percent.mt, and between nCount_RNA and nFeature_RNA in the filtered data; (C) Heatmap of the top 20 genes most correlated with PC_1–PC_6 in the PCA, where yellow indicates upregulation and purple indicate downregulation; (D) Left plot shows the distribution of cells in PC_1 and PC_2 before batch correction, with each dot representing a cell, and the right plot shows the violin plots of cells' distribution in PC_1 and PC_2; (E) Harmony batch correction process plot, with the horizontal axis representing the number of iterations; (F) Distribution of standard deviations of PCs, with more significant PCs having larger standard deviations. AP: n=2, control: n=2.

Supplementary Material 2. Figure S2. Cell Clustering of scRNA-seq Data. Note: (A) Distribution of cells in PC_1 and PC_2 after Harmony batch correction, with each dot representing a cell (left) and violin plots of the corrected data (right); (B) Clustering at different resolutions displayed using the Clustree package; (C) UMAP visualization of clustering results showing the aggregation and distribution of cells from Control (blue) and AP (red) samples in two dimensions; (D) UMAP visualization of clustering results, with each color representing a different cluster. AP: n=2, control: n=2.

Supplementary Material 3. Figure S3. Identification of Key Genes Involved in AP Progression in Macrophages. Note: (A) Venn diagram showing the overlap between scRNA-seq differential genes in macrophages and 18 characteristic genes from bulk RNA-seq; (B) UMAP plots showing the expression of ANXA3, PFKFB3, and SAMSN1 in the scRNA-seq dataset; (C) Violin plots of differential expression of ANXA3, PFKFB3, and SAMSN1 in macrophages. Bulk RNA-seq: AP: n=87, control: n=32; scRNA-seq: AP: n=2, control: n=2.

Supplementary Material 4.

## Data Availability

The data supporting the findings of this study are available from the public database Gene Expression Omnibus (GEO) (https://www.ncbi.nlm.nih.gov/geo/), including transcriptomic datasets such as single-cell RNA sequencing (scRNA-seq) and bulk RNA sequencing (bulk RNA-seq). Other data relevant to the conclusions of this study are available upon request from the corresponding author.

## Abbreviations

AP: Acute Pancreatitis
BP: Biological Processes
CC: Cellular Components
DEGs: Differentially Expressed Genes
DLS: Dynamic Light Scattering
ECAR: Extracellular Acidification Rate
GEO: Gene Expression Omnibus
GO: Gene Ontology
GSEA: Gene Set Enrichment Analysis
H&E: Hematoxylin and Eosin
HPLC: High-Performance Liquid Chromatography
KEGG: Kyoto Encyclopedia of Genes and Genomes
MAD: Median Absolute Deviation
MF: Molecular Functions
NTA: Nanoparticle Tracking Analyzer
OCR: Oxygen Consumption Rate
PCA: Principal Component Analysis
PCs: Principal Components
PFKFB3: 6-Phosphofructo-2-Kinase/Fructose-2,6-Bisphosphatase 3
Q-Ex: Quercetin-Loaded Extracellular Vesicles
RIPA: Radio Immunoprecipitation Assay
ROS: Reactive Oxygen Species
scRNA-seq: Single-Cell RNA Sequencing
SD: Sprague-Dawley
TEM: Transmission Electron Microscopy
WB: Western Blot
WGCNA: Weighted Gene Co-expression Network Analysis
