# Supplementary figures and images for: Targeting PFKFB3 to restore glucose metabolism in acute pancreatitis via nanovesicle delivery
Source: Mol Med. 2025 Jul 5;31:253. doi: 10.1186/s10020-025-01261-y (PMC12229013; doi:10.1186/s10020-025-01261-y)

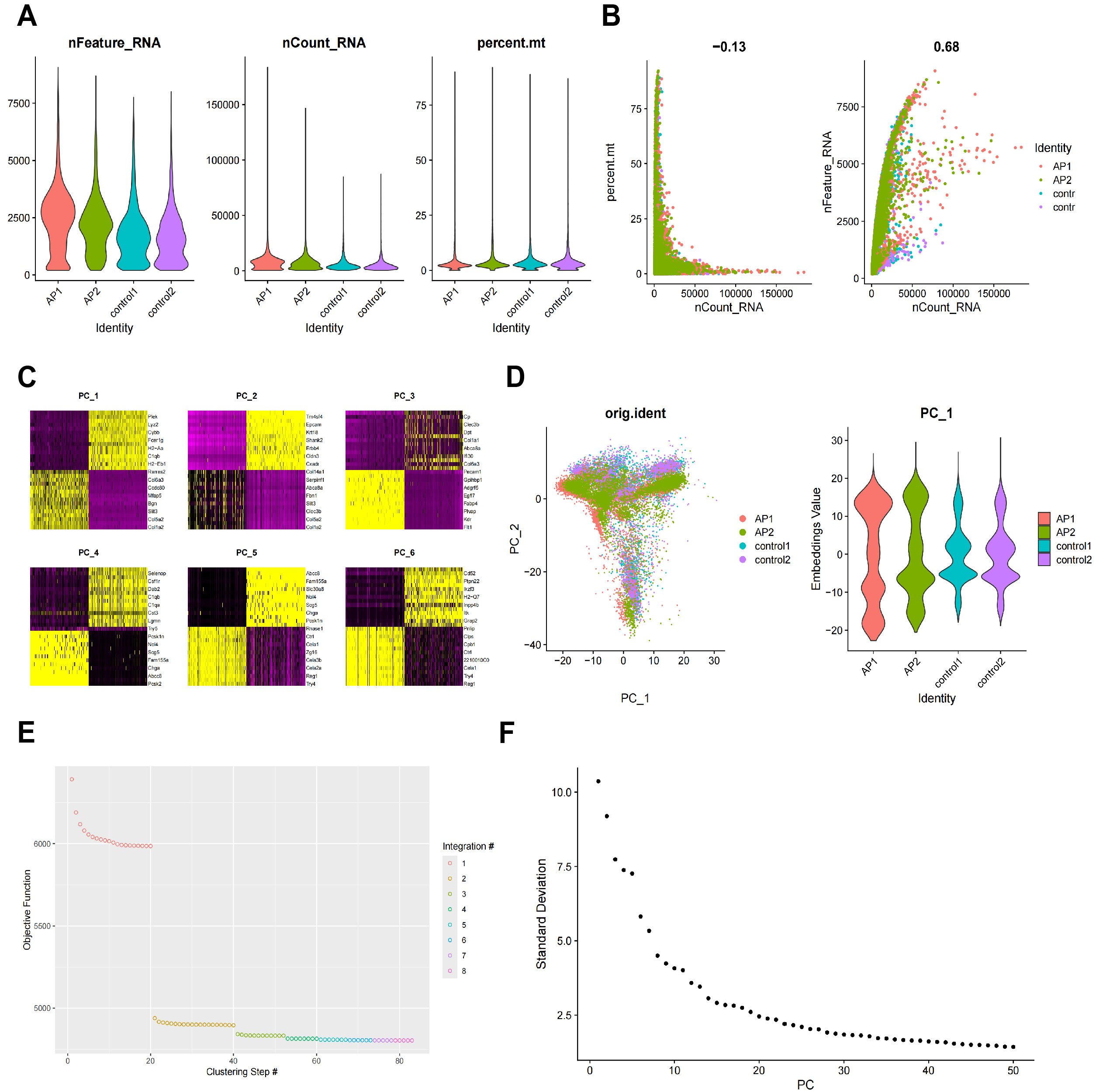

Supplement: Supplementary file 1 — Supplementary Material 1. Figure S1. Quality Control, Filtering, and PCA of scRNA-seq Data. Note: (A) Violin plots of the number of genes per cell (nFeature_RNA), the number of mRNA molecules per cell (nCount_RNA), and the percentage of mitochondrial genes (percent.mt) in the scRNA-seq data; (B) Scatter plots showing correlations between nCount_RNA and percent.mt, and between nCount_RNA and nFeature_RNA in the filtered data; (C) Heatmap of the top 20 genes most correlated with PC_1–PC_6 in the PCA, where yellow indicates upregulation and purple indicate downregulation; (D) Left plot shows the distribution of cells in PC_1 and PC_2 before batch correction, with each dot representing a cell, and the right plot shows the violin plots of cells' distribution in PC_1 and PC_2; (E) Harmony batch correction process plot, with the horizontal axis representing the number of iterations; (F) Distribution of standard deviations of PCs, with more significant PCs having larger standard deviations. AP: n=2, control: n=2. [file 10020_2025_1261_MOESM1_ESM.jpg]

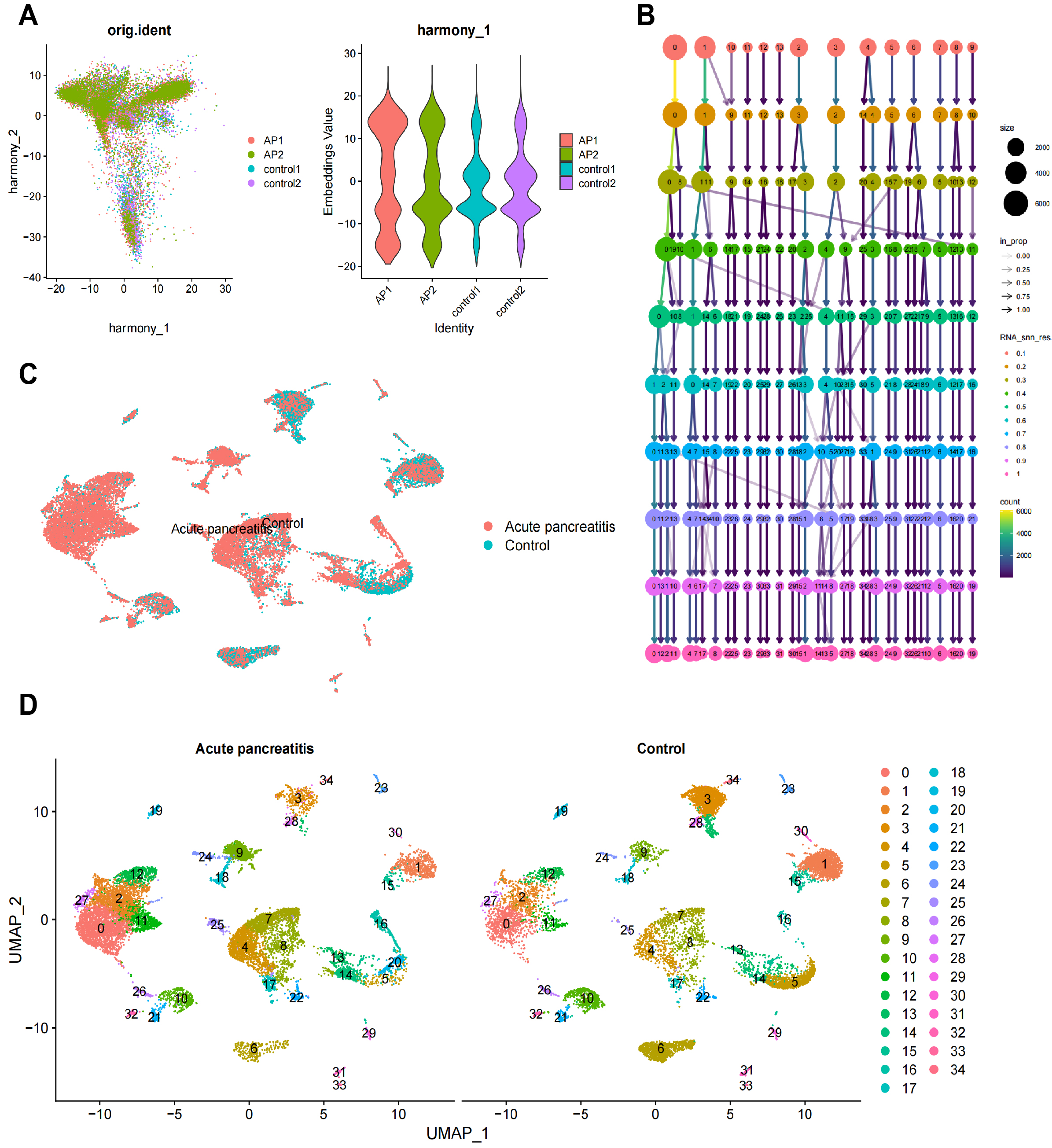

Supplement: Supplementary file 2 — Supplementary Material 2. Figure S2. Cell Clustering of scRNA-seq Data. Note: (A) Distribution of cells in PC_1 and PC_2 after Harmony batch correction, with each dot representing a cell (left) and violin plots of the corrected data (right); (B) Clustering at different resolutions displayed using the Clustree package; (C) UMAP visualization of clustering results showing the aggregation and distribution of cells from Control (blue) and AP (red) samples in two dimensions; (D) UMAP visualization of clustering results, with each color representing a different cluster. AP: n=2, control: n=2. [file 10020_2025_1261_MOESM2_ESM.jpg]

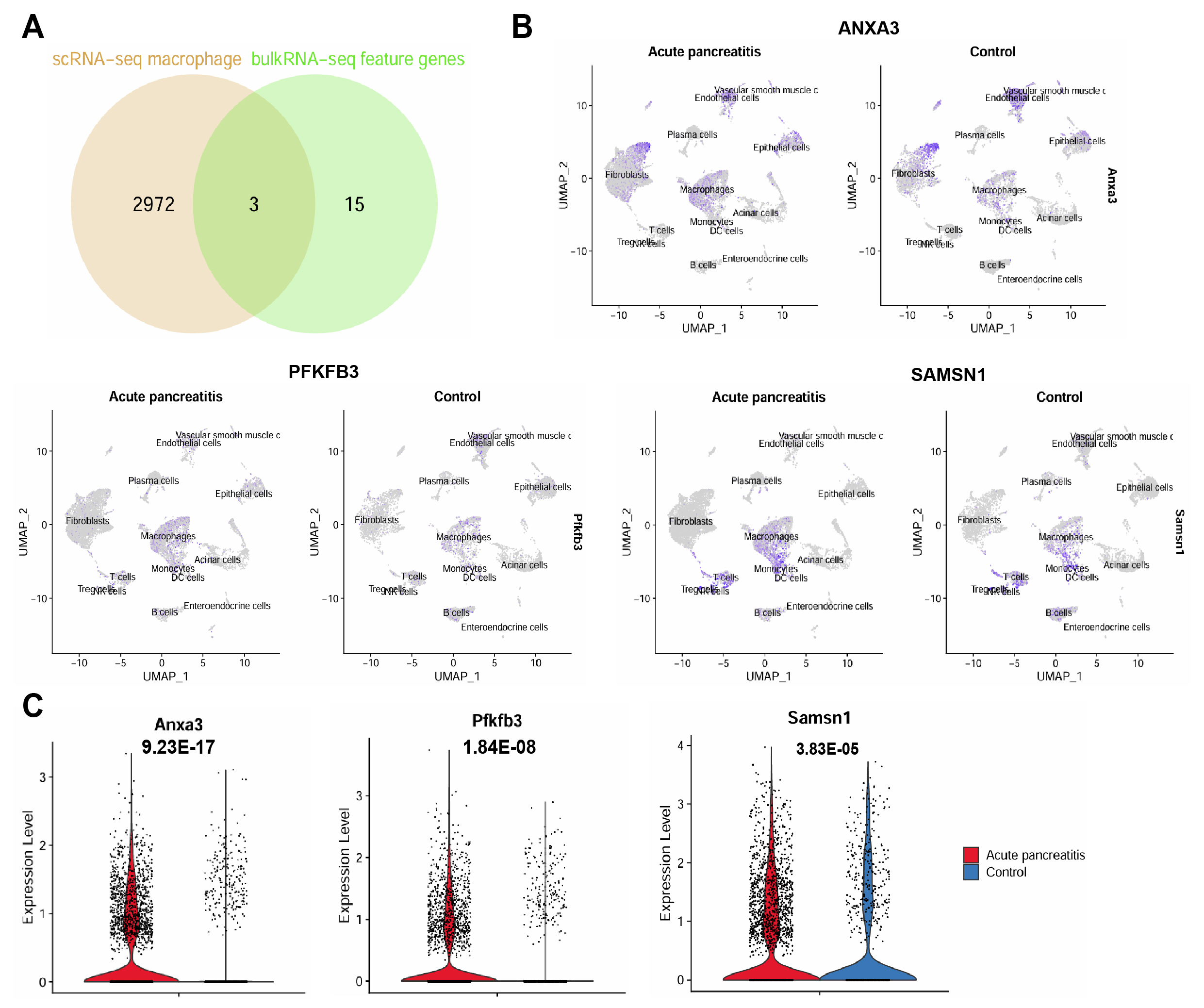

Supplement: Supplementary file 3 — Supplementary Material 3. Figure S3. Identification of Key Genes Involved in AP Progression in Macrophages. Note: (A) Venn diagram showing the overlap between scRNA-seq differential genes in macrophages and 18 characteristic genes from bulk RNA-seq; (B) UMAP plots showing the expression of ANXA3, PFKFB3, and SAMSN1 in the scRNA-seq dataset; (C) Violin plots of differential expression of ANXA3, PFKFB3, and SAMSN1 in macrophages. Bulk RNA-seq: AP: n=87, control: n=32; scRNA-seq: AP: n=2, control: n=2. [file 10020_2025_1261_MOESM3_ESM.jpg]
