# Supplementary material for: Targeting PFKFB3 to restore glucose metabolism in acute pancreatitis via nanovesicle delivery
Source: Mol Med. 2025 Jul 5;31:253. doi: 10.1186/s10020-025-01261-y (PMC12229013; doi:10.1186/s10020-025-01261-y)
Supplement: Supplementary file 4 — Supplementary Material 4. [file 10020_2025_1261_MOESM4_ESM.docx]

**Table S1. RT-qPCR Primer Sequences**

| **Gene Name** | **Primer Sequences** |
| --- | --- |
| IL-1β(mouse) | Forward: 5’- TGCCACCTTTTGACAGTGATG -3’ |
|  | Reverse: 5’- ATGTGCTGCTGCGAGATTTG -3’ |
| IL-6(mouse) | Forward: 5’- TTCCTCTGGTCTTCTGGAGT -3’ |
|  | Reverse: 5’- TGACTCCAGCTTATCTCTTGGTT -3’ |
| TNF-α(mouse) | Forward: 5’- ACCCTCACACTCACAAACCA -3’ |
|  | Reverse: 5’- ACCCTGAGCCATAATCCCCT -3’ |
| Pfkfb3(mouse) | Forward: 5’- CTGAAATGTCCGCTCCACAC -3’ |
|  | Reverse: 5’- AGCTCTTCATGTTCTCTGACCTC -3’ |
| Glut1(mouse) | Forward: 5’ - GCGGGAGACGCATAGTTACA -3’ |
|  | Reverse: 5’ - TGTTCATCGGTCTTGCTGCT -3’ |
| Glut3(mouse) | Forward: 5’- CAGCTCCAGCAAGCAATTCG -3’ |
|  | Reverse: 5’- GCTACCTCAAACACACCCGA -3’ |
| Ldha(mouse) | Forward: 5’- GTTGCTATGCCTTGGGGTCG -3’ |
|  | Reverse: 5’- TACAGCAAGTAGAGCGCCAA -3’ |
| Hk1(mouse) | Forward: 5’- GCTGCCTTCTTATGTTCGGA -3’ |
|  | Reverse: 5’- CAGTAGGACTCGGAAATTCGTT -3’ |
| Pfkfb3(rat) | Forward: 5’- CGGACAACCTTTGCTAGGGA -3’- |
|  | Reverse: 5’- TTCTGGGAAGATTCGGCACC -3’ |
| Glut1(rat) | Forward: 5’- GCTGTGGCTGGCTTCTCTAA -3’ |
|  | Reverse: 5’- CCGGAAGCGATCTCATCGAA -3’ |
| Glut3(rat) | Forward: 5’- GTCACTCCGGCGCTACG -3’ |
|  | Reverse: 5’- TTTCAAGGCACAGCTTCCATC -3’ |
| Ldha(rat) | Forward: 5’- GAGCTGTGGTTGGTCCAGTT -3’ |
|  | Reverse: 5’- GCAGTTGGCAGTGTGTCTTG -3’ |
| Hk1(rat) | Forward: 5’- CTCCAACCCACCAGCTCAAG -3’ |
|  | Reverse: 5’- TGCCATGCACGATGTTCTCT -3’ |
| GAPDH(mouse) | Forward: 5’- AAGAGGGATGCTGCCCTTAC -3’ |
|  | Reverse: 5’- TACGGCCAAATCCGTTCACA -3’ |
| GAPDH(rat) | Forward: 5’- CTCAGTTGCTGAGGAGTCCC -3’ |
|  | Reverse: 5’- ATTCGAGAGAAGGGAGGGCT -3’ |

**Table S2. Antibody Information and Dilution Ratios**

| **Gene** | **Company** | **NO.** | **Ratio** |
| --- | --- | --- | --- |
| IL-1 beta | Abcam | ab283818 | 1:1000 |
| IL-6 | Abcam | ab9324 | 1:1000 |
| TNF-α | Abcam | ab307164 | 1:1000 |
| PFKFB3 | Abcam | ab181861 | 1:1000 |
| GLUT1 | Abcam | ab115730 | 1:100000 |
| GLUT3 | Abcam | ab22048 | 1:1000 |
| HK1 | Abcam | ab150423 | 1:1000 |
| LDHA | Abcam | ab52488 | 1:5000 |
| β-actin | Abcam | ab8226 | 1:10000 |
